# HER2-targeted therapy prolongs survival in patients with HER2-positive breast cancer and intracranial metastatic disease: a systematic review and meta-analysis

**DOI:** 10.1093/noajnl/vdaa136

**Published:** 2020-10-14

**Authors:** Anders W Erickson, Farinaz Ghodrati, Steven Habbous, Katarzyna J Jerzak, Arjun Sahgal, Manmeet S Ahluwalia, Sunit Das

**Affiliations:** 1 Institute of Medical Science, Faculty of Medicine, University of Toronto, Toronto, Ontario, Canada; 2 Ontario Health (Cancer Care Ontario), Toronto, Ontario, Canada; 3 Division of Medical Oncology, Sunnybrook Health Sciences Centre, Toronto, Ontario, Canada; 4 Department of Radiation Oncology, Sunnybrook Hospital, Toronto, Ontario, Canada; 5 Burkhardt Brain Tumor and Neuro-Oncology Center, Cleveland Clinic, Cleveland, Ohio, USA; 6 Division of Neurosurgery, St. Michael’s Hospital, University of Toronto, Toronto, Ontario, Canada

**Keywords:** brain metastases, breast cancer, HER2/neu, molecular targeted therapy

## Abstract

**Background:**

Intracranial metastatic disease (IMD) is a serious and known complication of human epidermal growth factor receptor 2 (HER2)-positive breast cancer. The role of targeted therapy for patients with HER2-positive breast cancer and IMD remains unclear. In this study, we sought to evaluate the effect of HER2-targeted therapy on IMD from HER2-positive breast cancer.

**Methods:**

We searched MEDLINE, EMBASE, CENTRAL, and gray literature sources for interventional and observational studies reporting survival, response, and safety outcomes for patients with IMD receiving HER2-targeted therapy. We pooled outcomes through meta-analysis and examined confounder effects through forest plot stratification and meta-regression. Evidence quality was evaluated using GRADE (PROSPERO CRD42020161209).

**Results:**

A total of 97 studies (37 interventional and 60 observational) were included. HER2-targeted therapy was associated with prolonged overall survival (hazard ratio [HR] 0.47; 95% confidence interval [CI], 0.39–0.56) without significantly prolonged progression-free survival (HR 0.52; 95% CI, 0.27–1.02) versus non-targeted therapy; the intracranial objective response rate was 19% (95% CI, 12–27%), intracranial disease control rate 62% (95% CI, 55–69%), intracranial complete response rate 0% (95% CI, 0–0.01%), and grade 3+ adverse event rate 26% (95% CI, 11–45%). Risk of bias was high in 40% (39/97) of studies.

**Conclusion:**

These findings support a potential role for systemic HER2-targeted therapy in the treatment of patients with IMD from HER2-positive metastatic breast cancer.

**Key Points**

- We performed a meta-analysis of survival, response, and safety outcomes for 7157 patients from 97 studies.
- HER2-targeted therapy was associated with prolonged overall survival for patients with IMD from HER2-positive breast cancer.
- Our results support a potential role for systemic HER2-targeted therapy for this patient population.

**Importance of the Study**

We reviewed the literature and meta-analyzed outcomes for HER2-targeted therapy in patients with HER2-positive breast cancer and IMD. HER2-targeted therapy was associated with prolonged overall survival, notable response proportions, and an adverse event rate that may depend on drug structure. These findings support a potential role for HER2-targeted therapy in the treatment of IMD from HER2-positive metastatic breast cancer. Future trials should include patients with IMD to determine optimal treatment combinations and sequences and illuminate the role of novel therapies that may have efficacy in the central nervous system.

Intracranial metastatic disease (IMD) is one of the most feared complications of breast cancer, the most common cancer in women and the second most frequent cause of IMD, accounting for 15–20% of all brain metastases.^1–3^ Expression of the human epidermal growth factor receptor 2 (HER2) is associated with an increased risk of IMD (odds ratio 2.7; 95% confidence interval [CI], 2–3.7) compared to other breast cancer subtypes.^4,5^ Up to 50% of women with HER2-positive breast cancer develop IMD over their lifetime.^6–11^ Furthermore, the incidence of IMD in women with HER2-positive breast cancer is increasing due to advances in detection and improved systemic disease control.^12^ Diagnosis with IMD has significant implications for prognosis: the median survival for patients with HER2-positive metastatic breast cancer is 26.3–30 months with IMD versus 42.5–47.9 months without brain involvement.^11,13–15^ Furthermore, diagnosis with IMD may result in reduced quality of life because of neurological deficit, as well as a “loss of hope and a fear of loss of self.” ^3,16^

Treatment for IMD in patients with HER2-positive breast cancer has historically been limited to surgical resection and radiotherapy; the role for chemotherapy has generally been disappointing.^17–20^ The intracranial efficacy of chemotherapy is thought to be limited by cell-intrinsic resistance and poor penetration of drugs across the blood–brain barrier.^16,20^

The finding of prolonged survival with HER2 inhibition in women with HER2-positive metastatic breast cancer^21–25^ and the increased permeability of novel HER2 inhibitors into the brain^26^ have led to an interest in HER2-targeted therapy as a treatment of IMD from HER2-positive metastatic disease.^16,27^ Guidelines from the National Comprehensive Cancer Network,^18^ Congress of Neurological Surgeons,^17^ and European Association of Neuro-Oncology^19^ reflect the paucity of evidence to support or condemn the use of HER2-targeted therapy for IMD.

Although prior systemic reviews have been conducted, these studies do not speak to HER2 targeting agents developed since trastuzumab and lapatinib, and one is not restricted to patients with HER2-positive disease.^28,29^ Our understanding of outcomes among patients with HER2-positive breast cancer brain metastases who receive HER2-targeted therapy thus remains limited. To address this limitation, we conducted this systematic review and meta-analysis to update the literature on the effects of HER2-targeted therapy on survival, response, and safety outcomes in patients with HER2-positive breast cancer and IMD.

## Methods

### Eligibility Criteria

Included studies reported outcomes for patients with IMD from HER2-positive breast cancer who received post-IMD HER2-targeted therapy. Details are available in Supplementary Methods.

### Search Strategy

On January 27, 2020, we searched multiple databases and gray literature sources. The full search strategy is available in Supplementary Methods and Supplementary Tables S1–S3).

### Study Selection

Retrieved records underwent title-and-abstract review then full-text review. Independent reviewers (A.W.E. and F.G.) screened the studies in duplicate using the eligibility criteria (Supplementary Tables S4 and S5). Reasons for exclusion at full-text review were recorded. Disagreements were resolved by discussion. Cohen’s *κ* statistic was calculated for both steps.

### Data Extraction

Two independent reviewers (A.W.E. and F.G.) extracted all study outcomes and characteristics in duplicate. Disagreements were resolved through discussion. Only data specific to patients with IMD from HER2-positive breast cancer were extracted.

### Synthesis of Results

Principal summary measures were hazard ratios (HRs) for survival outcomes and proportions for response and safety outcomes. We estimated summary effect sizes through meta-analyses with random effects models using the inverse variance method. Tests for heterogeneity included *I*^2^, *τ*^2^, and *Q* statistics. Analysis was performed using the statistical programming language R (version 3.6.1, R Core Team, 2019)^30^ and the R packages robvis^31^ and meta.^32^

### Additional Analyses

We conducted subgroup and sensitivity analyses and meta-regression to estimate subgroup effect sizes, assess robustness, and investigate confounders (Supplementary Methods).

### Risk of Bias

We assessed risk of bias in randomized controlled trials (RCTs) using the Cochrane Risk of Bias (RoB 2) tool,^33^ cohort studies using the Newcastle–Ottawa Scale,^34^ and the one non-randomized controlled trial (NRCT) using the ROBINS-I tool.^35^ Independent reviewers (A.W.E. and F.G.) assessed risk in duplicate and resolved discrepancies through discussion. We assessed evidence quality using the Grading of Recommendations Assessment, Development and Evaluation (GRADE) framework,^36^ and publication bias through Egger’s test and funnel plot inspection.

## Results

**Figure 1.**
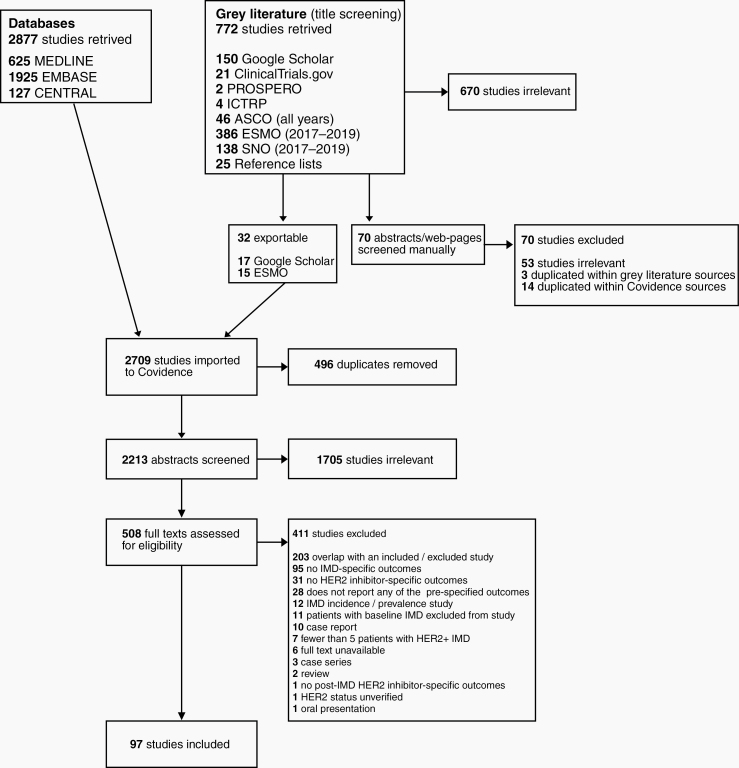
PRISMA flow diagram. Search queries were conducted in PubMed, EMBASE, CENTRAL, and gray literature source from their inception to January 27, 2020 for studies reporting survival, response, and safety outcomes for patients with IMD from HER2-positive breast cancer who received HER2-targeted therapy. Cohen’s *κ* statistic for inter-rater reliability at title-and-abstract (0.71) and full-text screening stages (0.67) indicated substantial agreement between reviewers.

The literature search yielded 3449 records, from which we included 97 studies and 7157 patients (Figure 1).^11,37–132^ The 97 included studies were 4 RCTs, 1 NRCT, 32 single-arm interventional trials, 1 prospective cohort study, and 59 retrospectives cohort studies. Thirty-six of the 41 comparative studies compared HER2-targeted therapy to a non-targeted therapy, and 5 compared different HER2-targeted therapies to one another. Median follow-up ranged from 6.25 to 26 months (Supplementary Table S6). Pharmaceutical industry funding was disclosed by 49% (48/97) of studies (Supplementary Table S7). Trial characteristics are listed in Table 1.

**Table 1.** Characteristics of Included Studies

Author	Year	Publication Type	Study Design	Therapy	Therapy (*n*)	Comparator	Comparator (*n*)
Chan, A. et al.^37^	2019	Abstr.	RCT	AC-TH or TCH	64	AC-T	37
Krop, I. et al.^38^	2015	Art.	RCT	T-DM1	45	Lapatinib + capecitabine	50
Murthy, R. et al.^39^	2019	Art.	RCT	Tucatinib + trastuzumab + capecitabine	198	Placebo + trastuzumab + capecitabine	93
Takano, T. et al.^40^	2018	Art.	RCT	Trastuzumab + capecitabine	6	Lapatinib + capecitabine	7
Bian, L. et al.^41^	2013	Art.	NRCT	Trastuzumab + capecitabine	4	Lapatinib + capecitabine	12
Brufsky, A. et al.^11^	2011	Art.	Pro. Coh.	Trastuzumab	258	No trastuzumab	119
Bartsch, R. et al.^42^	2011	Art.	Ret. Coh.	Trastuzumab ± lapatinib	43	No HER2-targeted therapy	37
Bartsch, R. et al.^43^	2007	Art.	Ret. Coh.	Trastuzumab	17	No trastuzumab	36
Braccini, A. et al.^44^	2013	Art.	Ret. Coh.	Trastuzumab and/or lapatinib	89	No HER2-targeted therapy	20
Chen, J. et al.^45^	2014	Abstr.	Ret. Coh.	HER2-targeted therapy	24	No HER2-targeted therapy	36
Church, D. et al.^46^	2008	Art.	Ret. Coh.	Trastuzumab	18	No trastuzumab	8
Gomes, D. et al.^47^	2015	Abstr.	Ret. Coh.	Trastuzumab and/or lapatinib	NR	No HER2-targeted therapy	NR
Gori, S. et al.^48^	2019	Art.	Ret. Coh.	Trastuzumab and/or lapatinib	102	No HER2-targeted therapy	52
Griguolo, G. et al.^49^	2018	Art.	Ret. Coh.	Pertuzumab, trastuzumab, T-DM1, and/or lapatinib	22	No HER2-targeted therapy	10
Hayashi, N. et al.^50^	2015	Art.	Ret. Coh.	Trastuzumab and/or lapatinib	283	No HER2-targeted therapy	149
Hulsbergen, A. et al.^51^	2020	Art.	Ret. Coh.	Trastuzumab and/or lapatinib	8	No HER2-targeted therapy	7
Kaplan, M. et al.^52^	2013	Art.	Ret. Coh.	Lapatinib + capecitabine	46	Trastuzumab-based therapy	65
Kaplan, M. et al.^53^	2015	Art.	Ret. Coh.	Trastuzumab ± lapatinib	20	No HER2-targeted therapy	30
Karam, I. et al.^54^	2011	Art.	Ret. Coh.	Trastuzumab + RT	130	RT	46
Kim, J. et al.^55^	2019	Art.	Ret. Coh.	Lapatinib + SRS	43	SRS	41
Le Scodan, R. et al.^56^	2011	Art.	Ret. Coh.	Trastuzumab	32	No trastuzumab	20
Metro, G. et al.^57^	2011	Art.	Ret. Coh.	Lapatinib + capecitabine	30	Trastuzumab-based therapy	23
Metro, G. et al.^58^	2007	Art.	Ret. Coh.	Trastuzumab	10	No trastuzumab	10
Miller, J. et al.^59^	2017	Art.	Ret. Coh.	Trastuzumab or lapatinib or pertuzumab or T-DM1	82	No HER2-targeted therapy	17
Morikawa, A. et al.^60^	2018	Art.	Ret. Coh.	Trastuzumab and/or lapatinib	80	No HER2-targeted therapy	20
Mounsey, L. et al.^61^	2018	Art.	Ret. Coh.	Trastuzumab, lapatinib, T-DM1, and/or pertuzumab	76	No HER2-targeted therapy	47
Mueller, V. et al.^62^	2016	Abstr.	Ret. Coh.	Trastuzumab or lapatinib or T-DM1 or Trastuzumab + pertuzumab	155	No HER2-targeted therapy	317
Niwinska, A. et al.^63^	2013	Abstr.	Ret. Coh.	Trastuzumab or lapatinib	NR	No HER2-targeted therapy	NR
Niwinska, A. et al.^64^	2010	Art.	Ret. Coh.	Trastuzumab and/or lapatinib	105	No HER2-targeted therapy	118
Okita, Y. et al.^65^	2013	Art.	Ret. Coh.	Trastuzumab	12	No trastuzumab	15
Ou, D. et al.^66^	2019	Art.	Ret. Coh.	HER2-targeted therapy	22	No HER2-targeted therapy	17
Park, I. et al.^67^	2009	Art.	Ret. Coh.	Trastuzumab	29	No trastuzumab	49
Park, Y. et al.^68^	2009	Art.	Ret. Coh.	Trastuzumab	40	No trastuzumab	37
Parsai, S. et al.^69^	2019	Art.	Ret. Coh.	Lapatinib + SRS	50	SRS	76
Tarhan, M. et al.^70^	2013	Art.	Ret. Coh.	Trastuzumab and/or lapatinib	21	No HER2-targeted therapy	15
Witzel, I. et al.^71^	2011	Art.	Ret. Coh.	Trastuzumab	NR	No trastuzumab	NR
Yap, Y. et al.^72^	2012	Art.	Ret. Coh.	Trastuzumab and/or lapatinib	115	No HER2-targeted therapy	165
Yomo, S. et al.^73^	2013	Art.	Ret. Coh.	Lapatinib + SRS	24	SRS	16
Zhang, C. et al.^74^	2016	Art.	Ret. Coh.	Trastuzumab	33	No trastuzumab	35
Zhang, Q. et al.^75^	2016	Art.	Ret. Coh.	Trastuzumab and/or lapatinib	24	No HER2-targeted therapy	36
Zhukova, L. et al.^76^	2018	Abstr.	Ret. Coh.	Trastuzumab ± lapatinib	NR	No HER2-targeted therapy	NR
Bhargava, P. et al.^77^	2019	Abstr.	Ret. Coh.	Lapatinib and/or trastuzumab or T-DM1 or trastuzumab (intrathecal)	102	—	NA
Bartsch, R. et al.^78^	2009	Art.	Ret. Coh.	Trastuzumab	40	—	NA
Bidard, F. et al.^79^	2009	Art.	Ret. Coh.	Trastuzumab ± lapatinib	6	—	NA
Fabi, A. et al.^80^	2018	Art.	Ret. Coh.	T-DM1	87	—	NA
Figura, N. et al.^81^	2019	Art.	Ret. Coh.	Trastuzumab (intrathecal)	18	—	NA
Gamucci, T. et al.^82^	2019	Art.	Ret. Coh.	Pertuzumab + trastuzumab + taxanes	21	—	NA
Gavila, J. et al.^83^	2019	Art.	Ret. Coh.	Trastuzumab + lapatinib	38	—	NA
Gori, S. et al.^84^	2012	Art.	Ret. Coh.	Trastuzumab	16	—	NA
Grell, P. et al.^85^	2012	Abstr.	Ret. Coh.	Lapatinib	31	—	NA
Hardy-Werbin, M. et al.^86^	2019	Art.	Ret. Coh.	T-DM1	5	—	NA
Huang, C. et al.^87^	2010	Abstr.	Ret. Coh.	Lapatinib + capecitabine	52	—	NA
Jackisch, C. et al.^88^	2014	Art.	Ret. Coh.	Trastuzumab	90	—	NA
Jacot, W. et al.^89^	2016	Art.	Ret. Coh.	T-DM1	39	—	NA
Mailliez, A. et al.^90^	2016	Abstr.	Ret. Coh.	T-DM1	14	—	NA
Martin Huertas, R. et al.^91^	2019	Abstr.	Ret. Coh.	T-DM1	8	—	NA
McCabe Y. et al.^92^	2016	Abstr.	Ret. Coh.	T-DM1	23	—	NA
Metro, G. et al.^93^	2010	Abstr.	Ret. Coh.	Trastuzumab + chemotherapy or ET	10	—	NA
Michel, L. et al.^94^	2015	Art.	Ret. Coh.	T-DM1	6	—	NA
Montagna, E. et al.^95^	2009	Art.	Ret. Coh.	Trastuzumab	36	—	NA
Okines, A. et al.^96^	2018	Art.	Ret. Coh.	T-DM1	16	—	NA
Riahi, H. et al.^97^	2010	Abstr.	Ret. Coh.	Trastuzumab + WBRT	31	—	NA
Rossi, M. et al.^98^	2016	Art.	Ret. Coh.	Trastuzumab	40	—	NA
Vasista, A. et al.^99^	2019	Art.	Ret. Coh.	Trastuzumab	29	—	NA
Vici, P. et al.^100^	2017	Art.	Ret. Coh.	T-DM1	61	—	NA
Bachelot, T. et al.^101^	2011	Art.	Sing. Int.	Lapatinib + capecitabine	45	—	NA
Bartsch, R. et al.^102^	2008	Art.	Sing. Int.	Trastuzumab + gemcitabine	5	—	NA
Bonneau, C. et al.^103^	2018	Art.	Sing. Int.	Trastuzumab (intrathecal)	16	—	NA
Borges, V. et al.^104^	2018	Art.	Sing. Int.	Tucatinib + T-DM1	30	—	NA
Christodoulou, C. et al.^105^	2017	Art.	Sing. Int.	Lapatinib + WBRT	12	—	NA
de Azambuja, E. et al.^106^	2013	Art.	Sing. Int.	Lapatinib + temozolomide	16	—	NA
Falchook, G. et al.^107^	2013	Art.	Sing. Int.	Trastuzumab + lapatinib + bevacizumab	10	—	NA
Freedman, R. et al.^108^	2019	Art.	Sing. Int.	Neratinib	40	—	NA
Giotta, F. et al.^109^	2010	Art.	Sing. Int.	Lapatinib + capecitabine	14	—	NA
Gutierrez, M. et al.^110^	2015	Abstr.	Sing. Int.	Trastuzumab (intrathecal)	19	—	NA
Hurvitz, S. et al.^111^	2018	Art.	Sing. Int.	Lapatinib + everolimus + capecitabine	19	—	NA
Leone, J. et al.^112^	2019	Art.	Sing. Int.	Trastuzumab + cabozantinib	21	—	NA
Lin, N. et al.^113^	2009	Art.	Sing. Int.	Lapatinib	242	—	NA
Lin, N. et al.^114^	2016	Abstr.	Sing. Int.	Pertuzumab + trastuzumab	40	—	NA
Lin, N. et al.^115^	2008	Art.	Sing. Int.	Lapatinib	39	—	NA
Lin, N. et al.^116^	2013	Art.	Sing. Int.	Lapatinib + WBRT + trastuzumab	35	—	NA
Lin, N. et al.^117^	2011	Art.	Sing. Int.	Lapatinib + capecitabine or topotecan	22	—	NA
MacPherson, I. et al.^118^	2019	Art.	Sing. Int.	Trastuzumab + epertinib or capecitabine	5	—	NA
Metzger, O. et al.^119^	2017	Abstr.	Sing. Int.	Tucatinib + trastuzumab	41	—	NA
Montemurro, F. et al.^120^	2017	Abstr.	Sing. Int.	T-DM1	399	—	NA
Morikawa, A. et al.^121^	2019	Art.	Sing. Int.	Lapatinib + capecitabine	11	—	NA
Murthy, R. et al.^122^	2018	Art.	Sing. Int.	Tucatinib ± capecitabine ± trastuzumab	29	—	NA
Naskhletashvili, D. et al.^123^	2010	Abstr.	Sing. Int.	Trastuzumab + capecitabine	5	—	NA
Niwinska, A. et al.^124^	2010	Art.	Sing. Int.	Trastuzumab + chemotherapy	52	—	NA
Pistilli, B. et al.^125^	2018	Art.	Sing. Int.	Trastuzumab + buparlisib + capecitabine	9	—	NA
Ro, J. et al.^126^	2012	Art.	Sing. Int.	Lapatinib + capecitabine	58	—	NA
Shawky, H. et al.^127^	2014	Art.	Sing. Int.	Lapatinib + capecitabine	21	—	NA
Sutherland, S. et al.^128^	2010	Art.	Sing. Int.	Lapatinib + capecitabine	34	—	NA
Toi, M. et al.^129^	2009	Art.	Sing. Int.	Lapatinib	10	—	NA
Van Swearingen, A. et al.^130^	2018	Art.	Sing. Int.	Trastuzumab + everolimus + vinorelbine	32	—	NA
Yardley, D. et al.^131^	2015	Art.	Sing. Int.	T-DM1	26	—	NA
Yardley, D. et al.^132^	2018	Art.	Sing. Int.	Lapatinib + cabazitaxel	11	—	NA

Art., article; Abstr., abstract; RCT, randomized controlled trial; NRCT, non-randomized controlled trial; Pro. Coh., prospective cohort study; Ret. Coh., retrospective cohort study; Sing. Int., single-arm interventional trial; AC-TH, doxorubicin + cyclophosphamide then trastuzumab + paclitaxel; TCH, paclitaxel + cyclophosphamide + trastuzumab; AC-T, doxorubicin + cyclophosphamide then paclitaxel; T-DM1, trastuzumab emtansine; RT, radiotherapy; SRS, stereotactic radiosurgery; —, none; NA, not applicable.

### Overall Survival

**Figure 2.**
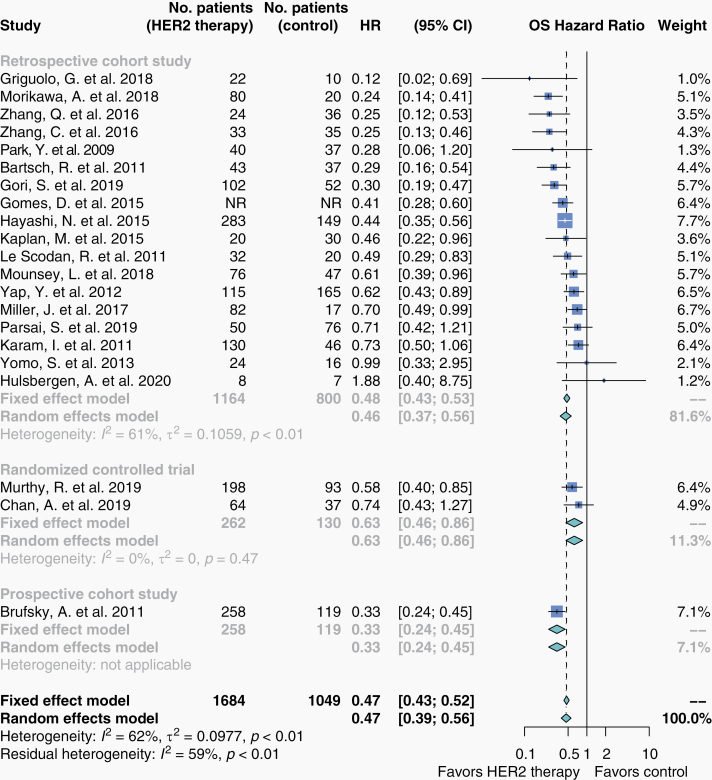
Overall survival in patients who received HER2-targeted therapy versus non-targeted therapy. Hazard ratios for overall survival were extracted from eligible studies and pooled in a meta-analysis. Studies here are stratified by study design. The size of each box represents the weight of each study in the meta-analysis. The vertical solid line represents the point of equivalence between HER2-targeted therapy and comparators. The vertical dashed and dotted lines represent the points of summary for fixed and random effects models, respectively, and the diamonds represent 95% CI. Analyses were performed with the R programming language^30^ and the R package meta.^32^

A meta-analysis of the 21 studies reporting overall survival (OS) HR comparing HER2-targeted therapy to non-targeted therapy showed HER2-targeted therapy was associated with prolonged OS (HR 0.47; 95% CI, 0.39–0.56; *n* = 3059; Figure 2). Summary estimates for individual agents for OS and all other outcomes are presented in Supplementary Table S8. Seventy-two studies reported OS in formats ineligible for meta-analysis (Supplementary Table S9).

### Progression-Free Survival

A meta-analysis of 4 studies showed that HER2-targeted therapy was not associated with prolonged progression-free survival (PFS; HR 0.52; 95% CI, 0.27–1.02; *n* = 475; Supplementary Figure S1). Twenty-nine studies reported PFS in formats ineligible for meta-analysis (Supplementary Table S10). Additional outcomes related to disease progression were reported in formats ineligible for meta-analysis: intracranial progression-free survival (iPFS), intracranial time to progression (iTTP), time to progression (TTP), and intracranial duration of response (iDoR) (Supplementary Tables S11–S14). Benefit with HER2-targeted therapy was seen in both studies reporting comparative iPFS (Supplementary Table S11) and in 3 of 4 studies reporting comparative iTTP (Supplementary Table S12). Comparative estimates for TTP and iDoR were not reported (Supplementary Tables S13 and S14).

### Intracranial Objective Response Rate

**Figure 3.**
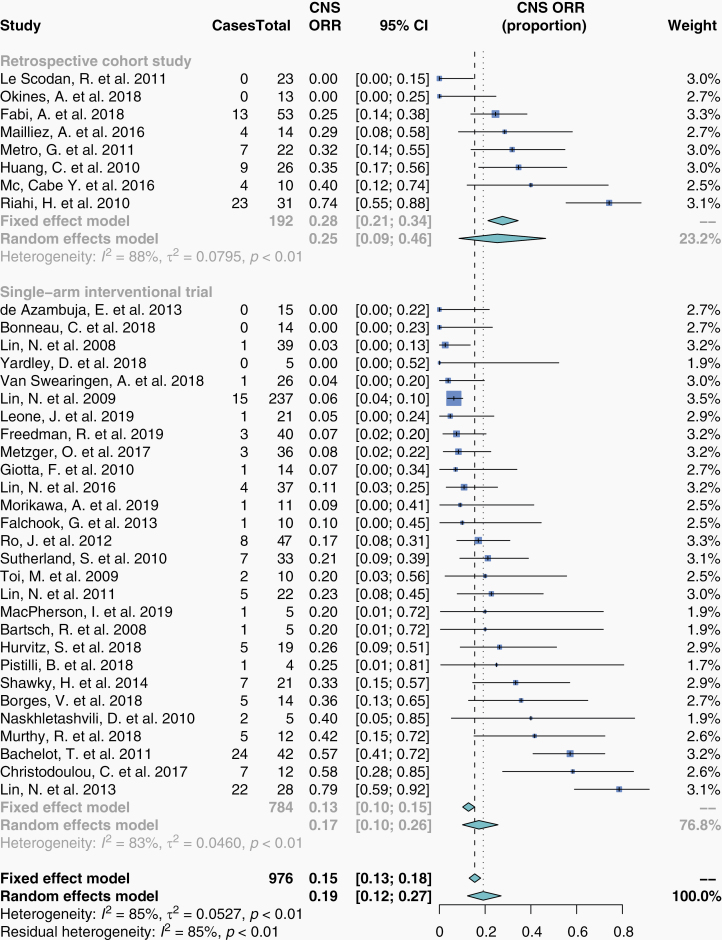
Intracranial objective response rate in patients who received HER2-targeted therapy. Proportions for iORR were extracted from eligible studies and pooled in a meta-analysis. Studies here are stratified by study design. The size of each box represents the weight of each study in the meta-analysis. The vertical dashed and dotted lines represent the points of summary for fixed and random effects models, respectively, and the diamonds represent 95% CI. Analyses were performed with the R programming language^30^ and the R package meta.^32^

We performed a meta-analysis for intracranial objective response rate (iORR) proportions from 36 studies. These were 28 single-arm interventional trials and 8 retrospective cohort studies. The summary estimate for iORR as a proportion was 19% (95% CI, 12–27%; *n* = 976; Figure 3).

### Intracranial Disease Control Rate

We performed a meta-analysis for intracranial disease control rate (iDCR) proportions from 33 studies. These were 1 NRCT, 25 single-arm interventional trials, and 7 retrospective cohort studies. The summary estimate for iDCR as a proportion was 62% (95% CI, 54–69%; *n* = 922; Supplementary Figure S2). Stratification by HER2-targeted agent and by publication before versus after 2018 produced distinct subgroup estimates and resolved some heterogeneity (Supplementary Figures S3 and S4).

### Intracranial Complete Response Rate

We then performed a meta-analysis on intracranial complete response rate (iCRR) proportions from 30 studies. These were 25 single-arm interventional trials and 5 retrospective cohort studies. The summary estimate for iCRR as a proportion was 0% (95% CI, 0–1%; *n* = 891; Supplementary Figure S5).

### Safety

**Figure 4.**
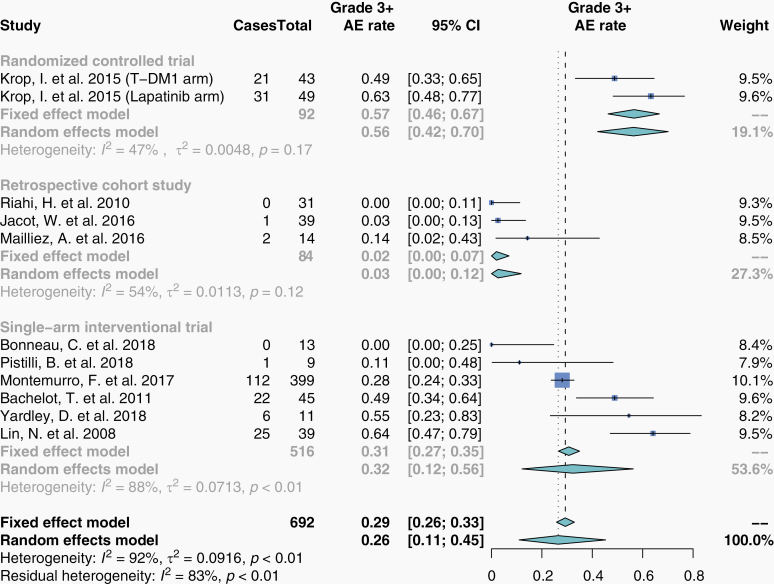
Grade 3+ CTCAE adverse event rate in patients who received HER2-targeted therapy. Proportions for grade 3+ CTCAE adverse event rate were extracted from eligible studies and pooled in a meta-analysis. Studies here are stratified by study design. The size of each box represents the weight of each study in the meta-analysis. The vertical dashed and dotted lines represent the points of summary for fixed and random effects models, respectively, and the diamonds represent 95% CI. Analyses were performed with the R programming language^30^ and the R package meta.^32^

Studies reported CTCAE grade 3+ adverse events as either a number of total events (15 studies; Supplementary Table S15) or as a number of patients who experienced events (10 studies; Figure 4). Summary estimate for grade 3+ adverse event rate from studies reporting patient numbers was 26% (95% CI, 11–45%; Figure 4). Stratification by drug structure (monoclonal antibody vs small-molecule inhibitor) produced distinct subgroup estimates and resolved some heterogeneity (Supplementary Figure S6). Only one study reported a central nervous system (CNS)-specific serious adverse event rate, which was 8% (30/399) in patients receiving T-DM1.^120^

### Additional Analyses

#### Sensitivity analyses

Sensitivity analyses did not produce significantly different summary estimates (Figures 2–4, Supplementary Figures S1, S2, and S5). Of note, omission of one study^37^ produced a significant summary estimate for PFS (HR 0.41; 95% CI, 0.30–0.56; *n* = 374).

#### Meta-regression

Meta-regression for OS, iORR, iDCR, and iCRR did not show association between selected characteristics and summary estimates (Supplementary Table S16). Two coefficients in the model for grade 3+ adverse event rate were significant: drug structure (small-molecule inhibitor vs monoclonal antibody, *β* = 0.33, *P* = .02) and study design (retrospective cohort study vs RCT, *β* = −0.47, *P* = .01).

### Risk of Bias

Risk of bias varied among the included studies (Supplementary Figures S7–S11). Egger’s test and visual inspection of funnel plots suggested asymmetry due to publication bias in the summary estimates for iDCR (*P* = .01, Supplementary Figure S12) and iCRR (*P* = .02, Supplementary Figure S13) and were undetected for other summary estimates (Supplementary Figures S14–S17).

### GRADE

Evidence certainty level differed between outcomes and study designs (Table 2).

**Table 2.** GRADE Summary of Findings

HER2-Targeted Therapy Compared To Control For Patients With Intracranial Metastatic Disease From HER2-Positive Breast Cancer											
Certainty Assessment							Summary of Findings				
Participants (studies) follow-up	Risk of bias	Inconsistency	Indirectness	Imprecision	Publication bias or effect size	Overall certainty of evidence	Study event rates (%)		Relative effect (95% CI)	Anticipated absolute effects	
							With control	With HER2- targeted therapy		Risk with control	Risk difference with HER2-targeted therapy
***Overall survival (OS), RCTs***											
392 (2 RCTs), follow-up NR	Not serious^a^	Not serious^b^	Not serious	Not serious	None	⨁⨁⨁⨁ HIGH	130 participants	262 participants	*HR 0.63* (0.46– 0.86) [OS]	*All patients*	
										50 per 100	*15 fewer per 100* (from 23 fewer to 5 fewer)
***OS, observational studies***											
2341 (19 observational studies), follow-up range 0.23–53 months	Serious^c^	Not serious^d^	Not serious	Not serious	Strong association^e^	⨁⨁◯◯ LOW	919 participants	1422 participants	*HR 0.45* (0.37– 0.54) [OS]	*All patients*	
										50 per 100	*23 fewer per 100* (from 27 fewer to 19 fewer)
***Progression-free survival, RCTs***											
392 (2 RCTs), follow-up NR	Not serious	Serious^f^	Not serious	Serious^g^	None	⨁⨁◯◯ LOW	130 participants	262 participants	*HR 0.74* (0.29–1.90) [Progression- free survival]	*All patients*	
										50 per 100	*10 fewer per 100* (from 32 fewer to 23 more)
***Progression-free survival, observational studies***											
83 (2 observational studies), follow-up range 1–39 months	Serious^h^	Not serious^b^	Not serious	Not serious	Strong association^i^	⨁⨁◯◯ LOW	42 participants	41 participants	*HR 0.32* (0.19 to 0.55) [Progression- free survival]	*All patients*	
										50 per 100	*30 fewer per 100* (from 38 fewer to 18 fewer)

CI, confidence interval; HR, hazard ratio.

^a^Low for both studies (RoB 2).

^b^ *I*-squared 0%, tau-squared 0.

^c^68% (13/19) studies Agency for Health Research and Quality (AHRQ) “poor.”

^d^ *I*-squared 63%, tau-squared 0.104.

^e^HR 0.45.

^f^ *I*-squared 89%, tau-squared 0.417.

^g^95% CI, 0.29–1.90.

^h^50% (1/2) studies AHRQ “poor.”

^i^HR 0.32.

## Discussion

In our meta-analysis, HER2-targeted therapy was associated with prolonged OS (HR 0.47; 95% CI, 0.39–0.56) in patients with HER2-positive breast cancer and IMD, with an iORR of 22% (95% CI, 14–30%), an iDCR of 62% (95% CI, 55–69%), an iCRR of 0% (95% CI, 0–0.01%), and a grade 3+ adverse event rate of 26% (95% CI, 11–45%). HER2-targeted therapy did not have a statistically significant effect on PFS (HR 0.52; 95% CI, 0.27–1.02).

The lack of prolonged PFS with HER2-targeted therapy may be an artifact of multiple data limitations. First, only 4 of 29 eligible studies included PFS data amenable to pooling. Second, the RECIST 1.1 criteria used to evaluate PFS do not distinguish between systemic and intracranial progression. Hence, a patient experiencing CNS benefit may be taken off therapy due to systemic progression. Third, this estimate was produced through pooling studies with different designs and treatments; this variety may both account for this result and reduce its credibility. Prolonged iPFS and iTTP were reported with HER2-targeted therapy versus non-targeted therapy by 2 and 3 studies, respectively (Supplementary Tables S11 and S12).

Subgroup analysis suggested that estimates for individual HER2-targeted agents were similar (Supplementary Table S8). Stratification of grade 3+ adverse event rate by drug structure suggested that antibody-based therapies were associated with lower rates of grade 3+ adverse events compared to small-molecule inhibitors (Supplementary Figure S6). This could be the result of greater pharmacokinetic distribution of small-molecule inhibitors compared to antibodies,^133,134^ an inherent difference in toxicity between classes or a spurious product of multiple comparisons.

Sensitivity analyses showed that our results were robust. Meta-regression revealed significant coefficients for study design and drug structure in modeling grade 3+ adverse event rate, although this analysis was underpowered due to the small ratio between the number of studies (*k* = 11) and model variables (*n* = 3).

Risk of bias varied in our study. Seventy-five percent (24/32) of single-arm interventional studies did not report central or blinded outcome measurement. Fifty-six percent (20/36) of comparative cohort studies either did not control or did not report control of confounders between study arms (Supplementary Figure S7). Most cohort studies did not report adequate follow-up (62%, 37/60) or follow-up completeness (82%, 49/60); Supplementary Figure S9).

Our results were consistent with previous reviews of trastuzumab and lapatinib for IMD from HER2-positive breast cancer.^28,29^ Reviews of other HER2-targeted therapies are lacking.

Since the execution of our literature search, the HER2CLIMB, CLEOPATRA, EMILIA, and KAMILLA trials have reported intracranial antitumor activity with the addition of tucatinib to trastuzumab and capecitabine, pertuzumab to trastuzumab plus docetaxel, T-DM1 versus lapatinib plus capecitabine, and T-DM1, respectively.^38,135–137^

Progress in the field of breast cancer brain metastases is still limited by infrequent evaluation of CNS-specific endpoints. This is reflected in the paucity of comparative intracranial results in our study: Of 36 studies comparing HER2-targeted therapy to a non-targeted comparator, none reported iORR, iDoR, iTTR, and iBCLS for both experimental and control arms, while only 1 trial reported iCRR, 2 reported iDCR and iPFS, and 4 evaluated iTTP. To obtain high-quality data regarding the efficacy of systemic therapy for the treatment of breast cancer patients with IMD, intracranial outcomes need to be collected prospectively in relevant RCTs. More liberal inclusion of patients with IMD should also be considered in the design of future clinical trials.^138–140^

### Limitations

Our study had several limitations. First, patients with IMD from HER2-positive breast cancer were a subgroup in many of the included studies and therefore, outcomes for these patients were often few and secondary. Second, heterogeneity was substantial or considerable in most of our summary estimates. This was expected as our study employed broad inclusion criteria. To resolve heterogeneity, our subgroup analyses and meta-regression identified important factors for several outcomes, although these may be false positives from multiple comparisons. Third, many outcomes were reported in formats that precluded meta-analysis. PFS, for example, was reported as an HR comparing HER2-targeted therapy to non-targeted therapy by only 4 of 29 studies reporting PFS. A more accurate approximation of effects could be achieved with increased reporting of meta-analyzable endpoints. Fourth, several outcomes key to clarifying the role of HER2-targeted therapy in the management of IMD were under-reported, such as comparative intracranial response and safety outcomes, and CNS-specific clinical features and mortality.

## Conclusions

Our study reviewed the literature and meta-analyzed outcomes for HER2-targeted therapy in patients with HER2-positive breast cancer and IMD. We find that HER2-targeted therapy is associated with prolonged OS, notable response proportions, and an adverse event rate that may depend on drug structure. Our findings support a role for HER2-targeted therapy in the treatment of IMD from HER2-positive metastatic breast cancer. Future trials for HER2-positive metastatic breast cancer should include patients with IMD to determine optimal treatment combinations and sequences, and further illuminate the role of novel therapies that may have efficacy in the CNS.

## Supplementary Material

vdaa136_suppl_Supplementary_Materials

## Notes

**Authorship statement**. Project design: A.W.E. and S.D.; literature search and screening: A.W.E. and F.G.; data extraction: A.W.E. and F.G.; statistical analysis: A.W.E. and F.G.; manuscript draft and editing: A.W.E., F.G., S.H., K.J.J., A.S., M.A., and S.D.; project supervision: S.D.
