# Supplementary material for: HER2-targeted therapy prolongs survival in patients with HER2-positive breast cancer and intracranial metastatic disease: a systematic review and meta-analysis
Source: Neurooncol Adv. 2020 Oct 14;2(1):vdaa136. doi: 10.1093/noajnl/vdaa136 (PMC7720818; doi:10.1093/noajnl/vdaa136)
Supplement: vdaa136_suppl_Supplementary_Materials [file vdaa136_suppl_supplementary_materials.docx]

**Supplement to HER2-targeted therapy for intracranial metastatic disease from breast cancer: a systematic review and meta-analysis**

Contents:

[Supplementary Methods 3](#_Toc46757339)

[Study records 3](#_Toc46757340)

[Data management 3](#_Toc46757341)

[Selection process 3](#_Toc46757342)

[Data collection process 3](#_Toc46757343)

[Data items 3](#_Toc46757344)

[Pre-processing 3](#_Toc46757345)

[Data extraction 4](#_Toc46757346)

[GRADE summary of findings table 4](#_Toc46757347)

[Supplementary Tables 5](#_Toc46757348)

[Table S1. MEDLINE search query 5](#_Toc46757349)

[Table S2. EMBASE search query 6](#_Toc46757350)

[Table S3. CENTRAL search query 7](#_Toc46757351)

[Table S4. Inclusion/exclusion criteria for abstract/title screening 9](#_Toc46757352)

[Table S5. Inclusion/exclusion criteria for full-text review 11](#_Toc46757353)

[Table S6. Median follow-up 12](#_Toc46757354)

[Table S7. Pharmaceutical industry funding among reporting studies. 13](#_Toc46757355)

[Table S8. Summary estimates for individual HER2-targeted agents. 17](#_Toc46757356)

[Table S9. Overall survival (OS) as reported in months 19](#_Toc46757357)

[Table S10. Progression-free survival (PFS) as reported in months 23](#_Toc46757358)

[Table S11. Intracranial progression-free survival (iPFS) as reported in months 25](#_Toc46757359)

[Table S12. Time to intracranial progression (iTTP) as reported in months 26](#_Toc46757360)

[Table S13. Time to progression (TTP) as reported in months 27](#_Toc46757361)

[Table S14. Intracranial duration of response (iDoR) as reported in months 28](#_Toc46757362)

[Table S15. Frequency of Grade 3+ CTCAE adverse events (AEs), reported as numbers of events 29](#_Toc46757363)

[Table S16. Results of meta-regression analyses for 3-variable models including drug structure, publication year, and study design. 30](#_Toc46757364)

[Supplementary Figure Legends 31](#_Toc46757365)

[References 35](#_Toc46757366)

## Supplementary Methods

The current study was conducted in accordance with the Preferred Reporting Items for Systematic Reviews and Meta-Analyses (PRISMA) guidelines^1^ and was registered on PROSPERO (CRD42020161209) and Open Science Framework (doi: 10.17605/OSF.IO/EZCM6).

#### Data management

Search results were exported into EndNote for storage. Results were uploaded to Covidence, and duplicates removed using that software’s native duplicate removal feature. Remaining duplicates were manually removed.

#### Eligibility criteria

Included study designs were randomized controlled trials (RCTs), non-randomized controlled trials (NRCTs), and cohort studies. Case-control studies, cross-sectional studies, case reports, case series and reviews were excluded. Search results were limited to the English language. There were no restrictions by study setting, length of follow-up, publication year, or publication format (article or abstract). We assessed study eligibility using the PICO approach.

**Population:** adult patients age 18+ with IMD from HER2-positive breast cancer.

**Intervention:** HER2-targeted therapy, including monotherapy or in combination with other treatment modalities, and as first-line therapy or any line of therapy. HER2-targeted therapies of interest were: trastuzumab, ado-trastuzumab emtansine (T-DM1), pertuzumab, neratinib, lapatinib, tucatinib, ertumaxomab, pyrotinib, epertinib, SYD-985 and DS-8201.

**Comparison:** no restrictions.

**Outcome:** Pre-specified outcomes included: overall survival (OS), progression-free survival (PFS), intracranial progression-free survival (iPFS), intracranial objective response rate (iORR), intracranial disease control rate (iDCR), intracranial complete response rate (iCRR), intracranial duration of response (iDoR), time to intracranial response (iTTR), best change in intracranial lesion size (iBCLS), and grade 3+ Common Terminology Criteria for Adverse Events (CTCAE) adverse event rate. We extracted time to progression (TTP) and time to intracranial progression (iTTP) post-hoc. Outcome definitions are presented in the Supplementary Methods.

#### Search strategy

We conducted searches in MEDLINE (OVID interface, all available years), EMBASE (OVID interface, all available years), and the Cochrane Central Register of Controlled Trials (CENTRAL, Wiley interface, current issue). We also scanned the reference lists of included or relevant studies. Grey literature sources included ClinicalTrials.gov, Google Scholar (first 150 results), PROSPERO, the International Clinical Trials Registry Platform (ICTRP), and the home pages of the Society for Neuro-oncology (SNO), the American Society of Clinical Oncology (ASCO), and the European Society of Medical Oncology (ESMO). Grey literature sources were hand searched using combinations of the following keywords: “breast cancer” and “HER2” and “brain metastases” and “CNS metastases” and “targeted therapy” and screened by title for relevant abstracts or records.

#### Selection process

In cases where a study of patients with breast cancer had published a separate subgroup analysis of patients with brain metastases from breast cancer, data from the subgroup analysis publication was used, and data from the main analysis was not used. Similarly, in cases where a study of patients with breast cancer brain metastases had published a separate subgroup analysis of patients specifically with HER2-positive disease, data from the HER2-positive subgroup analysis publication was used, and data from the main analysis was not used.

Reviewers piloted the screening criteria on a test set of 100 publications, and discussed the results. Inter-rater agreement for the test set was 85%, corresponding to κ 0.48, indicating moderate agreement. Following this, reviewers amended the screening questions and eligibility criteria for clarity. The final eligibility criteria were utilized on the full set of collected records and reported in this supplement. None of the selection process was blinded.

#### Data collection process

A charting form was prospectively developed by reviewers as a Google Sheets spreadsheet to determine which variables to extract, with definitions for each variable. Data extraction was piloted on a small subset of articles to improve consistency between authors. Then, two reviewers (AE, FG) independently extracted data in duplicate from included articles. A central data extraction spreadsheet was then populated.

#### Data items

In cases where multiple extractable values were available for a single outcome (e.g. if hazard ratios (HR) are available for OS for 1) HER2-targeted antibodies, and 2) HER2-targeted TKIs, to avoid counting patients twice who may have received both classes of therapy, the value with a larger sample size was used in quantitative synthesis. Remaining values were presented in tables.

*Outcome definitions*

| **Outcome** | **Definition** |
| --- | --- |
| Overall survival (OS) | The length of time from diagnosis of IMD until death or loss to follow up (censored). |
| Progression-free survival (PFS) | The length of time from diagnosis of IMD until disease progression, death, or loss to follow up (censored), where progression is defined as 30% increase or more in the sum of target lesions, per RECIST 1.1 criteria.[^4^](https://paperpile.com/c/6S45Z5/RQgi) |
| Progression-free survival, intracranial (iPFS) | The length of time from diagnosis of IMD until intracranial disease progression, death, or loss to follow up (censored), where intracranial progression is defined as 30% increase or more in the sum of intracranial target lesion largest diameters, per RECIST 1.1 criteria.[^4^](https://paperpile.com/c/6S45Z5/RQgi) |
| Disease control rate, intracranial (iDCR) | The percentage of patients whose intracranial disease responds or remains stable following treatment i.e. the sum of intracranial stable disease rate + complete intracranial response rate + partial intracranial response rate. |
| Objective response rate, intracranial (iORR) | The percentage of patients whose intracranial disease responds following treatment i.e. the sum of complete intracranial response rate + partial intracranial response rate. |
| Complete response rate, intracranial (iCRR) | The percentage of patients whose intracranial disease completely disappears following treatment, per RECIST 1.1 criteria.[^4^](https://paperpile.com/c/6S45Z5/RQgi) |
| Duration of response, intracranial (iDoR) | The length of time from documented intracranial complete response or intracranial partial response until intracranial disease progression, but not death or loss to follow up. |
| Time to response, intracranial (iTTR) | The length of time from first treatment until documented intracranial complete response or intracranial partial response. |
| Best change in lesion size, intracranial (iBCLS) | The largest documented percentage changes in intracranial lesion size from baseline documentation. |
| Grade 3+ adverse event rate | Adverse events greater than or equal to grade 3 according to Common Terminology Criteria for Adverse Events (CTCAE).[^5^](https://paperpile.com/c/6S45Z5/5h3i) |
| CNS adverse event rate | Rate of reported neurological adverse events per CTCAE: headache, fatigue, nausea, vomiting, dizziness, numbness, paresthesia.[^5^](https://paperpile.com/c/6S45Z5/5h3i) |

#### Data extraction

Our study did not distinguish between outcomes specific to patients who initiated a course of HER2-targeted therapy (not necessarily their first) following diagnosis of IMD, versus patients who developed IMD while on a course of HER2-targeted therapy and continued that course following IMD diagnosis.

Extracted trial characteristics included: author names, publication year, study design, treatment and comparator regimens, sample size, publication format (abstract vs. article), line-of-therapy, pharmaceutical industry funding, whether the HER2-targeted therapy was delivered as a monotherapy, whether the majority of patients had leptomeningeal metastases, and whether patients with IMD from HER2-positive breast cancer were a study subgroup or the study population.

#### Pre-processing

Proportional outcomes were transformed prior to meta-analysis depending on several conditions. If the distribution of a proportion was approximately normal, determined by histogram and Q-Q plot, and lacked values < 0.2 or > 0.8, then it was meta-analyzed as a raw proportion without transformation. Outcomes with proportions < 0.2 or > 0.8, and no values equal to zero, underwent logit transformation prior to meta-analysis. Outcomes with any proportions equal to zero underwent double-arcsine transformation. These were performed using the summary method argument in the R function metaprop in the package meta.^2^

#### Synthesis of results

We synthesized extracted data in several ways depending on comparator regimen. Results from studies comparing HER2-targeted therapy to non-targeted standard therapy (systemic chemotherapy, immunotherapy, surgery, whole-brain radiotherapy, or stereotactic radiosurgery) were synthesized through generic meta-analysis. Results from studies with no comparator arm were synthesized through meta-analysis of single proportions. Results from studies comparing different HER2-targeted therapies (e.g. trastuzumab vs. lapatinib) were treated as individual single-arm studies and synthesized through meta-analysis of single proportions. Experimental and comparator arms from studies comparing the same HER2-targeted therapy to itself (e.g. trastuzumab plus taxane vs. trastuzumab plus anthracycline) were combined as one single-arm study (trastuzumab plus chemotherapy) and synthesized through meta-analysis of single proportions.

We employed *I*^2^ ranges of 0–40%, 30–60%, 50–90%, and 75–100% to benchmark values for potentially unimportant, moderate, substantial, and considerable heterogeneity.^3^ The restricted maximum likelihood estimator was used for τ^2^.

#### Missing data

One missing value for OS HR was imputed using the method by Guyot et al.^4^ in one study^5^ where a stratified Kaplan-Meier plot was presented with an at-risk table. We did not contact study authors to retrieve missing data.

#### Additional analyses

We conducted subgroup analyses through forest plot stratification by study characteristics to estimate subgroup effect sizes and investigate heterogeneity in summary estimates. For subgroup estimates for individual HER2-targeted agents, a study was allowed to contribute if its treatment arm involved any receipt of that agent, and the comparator arm, if present, did not. For example, a study of “trastuzumab ± lapatinib” versus a comparator containing neither therapy would contribute to subgroup estimates for each of trastuzumab and lapatinib.

To assess summary estimate robustness, we conducted three sensitivity analyses. First, we estimated summary effect sizes using fixed-effects models.^6,7^ Second, we omitted studies at high risk of bias. Third, we iteratively omitted each study in a “leave-out-one” analysis to evaluate whether any single study substantially influenced the results.^7^

To adjust summary estimates for potential confounders, we conducted post-hoc metaregression for each summary estimate using a three-variable model based on key pre-specified study characteristics: study design, publication year, and drug structure (antibody-based vs. small-molecule inhibitor).

#### Risk of bias assessment

Newcastle Ottawa Scale (NOS) scores were converted to Agency for Health Research and Quality (AHRQ) standards “good,” “fair,” and “poor.” One year was considered “adequate follow-up” in the NOS based on the diagnosis-specific graded prognostic assessment for IMD from HER2-positive breast cancer.^8^

#### GRADE summary of findings table

Anticipated absolute effects for risk with HER2-targeted therapy for OS and PFS were calculated automatically in the GRADEpro software^9^ using corresponding summary HRs and manually designated 50% proportions in the control groups, corresponding to timepoints of median survival. Baseline certainty levels were set to “high” and “low” for RCTs and observational studies, respectively.^10^

##

## Supplementary Tables

### Table S1. MEDLINE search query

Jan 27, 2020

Database(s): Ovid MEDLINE: Epub Ahead of Print, In-Process & Other Non-Indexed Citations, Ovid MEDLINE® Daily and Ovid MEDLINE® 1946-Present

| # | Searches | Results |
| --- | --- | --- |
| 1 | exp Central Nervous System Neoplasms/ | 180404 |
| 2 | exp Cerebral Cortex/ | 353554 |
| 3 | exp Brain/ | 1186939 |
| 4 | exp blood-brain barrier/ | 26961 |
| 5 | 1 or 2 or 3 or 4 | 1325116 |
| 6 | exp Neoplasm Metastasis/ | 199612 |
| 7 | 5 and 6 | 6893 |
| 8 | exp Central Nervous System Neoplasms/sc | 17346 |
| 9 | ((brain* or intra?cranial or cerebral or cerebrum or crani* or skull or cns or leptomening* or mening* or posterior fossa or frontal lobe or parietal lobe or temporal lobe or occipital lobe or insula* or cortex or cortic* or encephal* or hippocamp* or gyrus or limbic or dentate or white matter or gr$y matter) adj3 (metasta* or (secondar* adj3 (malig* or cancer or disease or neoplas* or tumo?r* or carcinoma* or spread*)))).mp. | 21425 |
| 10 | 7 or 8 or 9 | 32263 |
| 11 | exp Breast/ | 45544 |
| 12 | exp Breast Diseases/ | 302537 |
| 13 | 11 or 12 | 319991 |
| 14 | exp Neoplasms/ | 3267067 |
| 15 | 13 and 14 | 290087 |
| 16 | exp Breast Neoplasms/ | 285869 |
| 17 | exp carcinoma, ductal, breast/ | 15476 |
| 18 | exp carcinoma, lobular/ | 5458 |
| 19 | ((breast* or mammary*) adj3 (neoplas* or cancer* or carcin* or tumo?r* or metasta* or malig*)).mp. | 403741 |
| 20 | 15 or 16 or 17 or 18 or 19 | 407252 |
| 21 | (((target* or molecular*) adj3 (therap* or agent* or drug* or inhib*)) and (HER2* or HER-2*)).mp. | 6550 |
| 22 | exp Receptor, ErbB-2/ | 23708 |
| 23 | exp molecular targeted therapy/ | 26698 |
| 24 | exp Antineoplastic Agents/ | 1082925 |
| 25 | exp antibodies, monoclonal/ | 230105 |
| 26 | exp protein kinase inhibitors/ | 93715 |
| 27 | exp antineoplastic protocols/ | 137205 |
| 28 | 22 and (23 or 24 or 25 or 26 or 27) | 10177 |
| 29 | ((HER2* or HER-2* or ERBB2* or ERB-B2* or CD340* or MLN19* or NEU or NGL* or TKR1*) adj3 (inhib* or therap* or agent* or drug*)).mp. | 5321 |
| 30 | exp trastuzumab/ | 6600 |
| 31 | exp lapatinib/ | 1506 |
| 32 | (trastuzumab* or ado-trastuzumab* or T-DM1* or pertuzumab* or lapatinib* or neratinib* or tucatinib* or ertumaxomab* or pyrotinib* or epertinib* or DS-8201* or DS8201* or SYD-985* or SYD985*).mp. | 12726 |
| 33 | 21 or 28 or 29 or 30 or 31 or 32 | 22056 |
| 34 | 10 and 20 and 33 | 625 |

###

### Table S2. EMBASE search query

Jan 27, 2020

Database(s): Embase Classic+Embase 1947 to 2020 January 27

| # | Searches | Results |
| --- | --- | --- |
| 1 | central nervous system metastasis/ or exp brain metastasis/ or exp meningeal metastasis/ | 36337 |
| 2 | ((brain* or intra?cranial or cerebral or cerebrum or crani* or skull or cns or leptomening* or mening* or posterior fossa or frontal lobe or parietal lobe or temporal lobe or occipital lobe or insula* or cortex or cortic* or encephal* or hippocamp* or gyrus or limbic or dentate or white matter or gr$y matter) adj3 (metasta* or (secondar* adj3 (malig* or cancer or disease or neoplas* or tumo?r* or carcinoma* or spread*)))).tw,kw. | 36142 |
| 3 | 1 or 2 | 48268 |
| 4 | breast tumor/ or exp breast cancer/ | 535604 |
| 5 | ((breast* or mammary*) adj3 (neoplas* or cancer* or carcin* or tumo?r* or metasta* or malig*)).tw,kw,sh. | 593860 |
| 6 | 4 or 5 | 637211 |
| 7 | exp epidermal growth factor receptor kinase inhibitor/ | 118724 |
| 8 | (HER-2* or HER2*).tw,kw,sh. | 62386 |
| 9 | 7 and 8 | 18984 |
| 10 | ((HER-2* or HER2* or ERBB2* or ERB-B2* or CD340* or NEU or MLN19* or NGL* or TKR1*) adj3 (inhib* or therap* or agent* or drug*)).tw,kw,sh. | 10795 |
| 11 | exp trastuzumab deruxtecan/ or exp trastuzumab/ or exp trastuzumab emtansine/ or exp trastuzumab duocarmazine/ | 38045 |
| 12 | exp lapatinib/ or exp lapatinib plus pazopanib/ | 11662 |
| 13 | exp pertuzumab/ | 4617 |
| 14 | exp neratinib/ | 1501 |
| 15 | exp tucatinib/ | 93 |
| 16 | exp ertumaxomab/ | 123 |
| 17 | exp epertinib/ | 7 |
| 18 | (trastuzumab* or ado-trastuzumab* or T-DM1* or pertuzumab* or lapatinib* or neratinib* or tucatinib* or ertumaxomab* or pyrotinib* or epertinib* or DS-8201* or DS8201* or SYD-985* or SYD985*).tw,kw,sh. | 45870 |
| 19 | 9 or 10 or 11 or 12 or 13 or 14 or 15 or 16 or 17 or 18 | 51273 |
| 20 | 3 and 6 and 19 | 1925 |

###

### Table S3. CENTRAL search query

Jan 27, 2020

| # | Searches | Results |
| --- | --- | --- |
| 1 | [mh "Central Nervous System Neoplasms"] | 2113 |
| 2 | [mh "Cerebral Cortex"] | 4654 |
| 3 | [mh Brain] | 11731 |
| 4 | [mh "blood-brain barrier"] | 122 |
| 5 | #1 or #2 or #3 or #4 | 13619 |
| 6 | [mh "Neoplasm Metastasis"] | 4854 |
| 7 | #5 and #6 | 123 |
| 8 | MeSH descriptor: [Central Nervous System Neoplasms] explode all trees and with qualifier(s): [secondary - SC] | 352 |
| 9 | ((brain* or intra*cranial or cerebral or cerebrum or crani* or skull or cns or leptomening* or mening* or posterior fossa or frontal lobe or parietal lobe or temporal lobe or occipital lobe or insula* or cortex or cortic* or encephal* or hippocamp* or gyrus or limbic or dentate or white matter or gr*y matter) NEAR/3 (metasta* or (secondar* NEAR/3 (malig* or cancer or disease or neoplas* or tumo*r* or carcinoma* or spread*)))):ti,ab | 2159 |
| 10 | #7 or #8 or #9 | 2289 |
| 11 | [mh Breast] | 726 |
| 12 | [mh "Breast Diseases"] | 12390 |
| 13 | #11 or #12 | 12751 |
| 14 | [mh Neoplasms] | 72725 |
| 15 | #13 and #14 | 12087 |
| 16 | [mh "Breast Neoplasms"] | 12044 |
| 17 | [mh "Mammary Ductal Carcinoma"] | 346 |
| 18 | [mh "Lobular Carcinoma"] | 157 |
| 19 | ((breast* or mammary*) NEAR/3 (neoplas* or cancer* or carcin* or tumo*r* or metasta* or malig*)):ti,ab | 33032 |
| 20 | #15 or #16 or #17 or #18 or #19 | 34117 |
| 21 | (((target* or molecular*) NEAR/3 (therap* or agent* or drug* or inhib*)) and (HER2* or HER-2*)):ti,ab | 536 |
| 22 | [mh "ErbB-2 Receptor"] | 725 |
| 23 | [mh "molecular targeted therapy"] | 135 |
| 24 | [mh "Antineoplastic Agents"] | 11771 |
| 25 | [mh "monoclonal antibodies"] | 11064 |
| 26 | [mh "protein kinase inhibitors"] | 780 |
| 27 | [mh "antineoplastic protocols"] | 13277 |
| 28 | #22 and (#23 or #24 or #25 or #26 or #27) | 622 |
| 29 | ((HER2* or HER-2* or ERBB2* or ERB-B2* or CD340* or MLN19* or NEU or NGL* or TKR1*) NEAR/3 (inhib* or therap* or agent* or drug*)):ti,ab | 804 |
| 30 | [mh trastuzumab] | 563 |
| 31 | [mh lapatinib] | 192 |
| 32 | (trastuzumab* or ado-trastuzumab* or T-DM1* or pertuzumab* or lapatinib* or neratinib* or tucatinib* or ertumaxomab* or pyrotinib* or epertinib* or DS-8201* or DS8201* or SYD-985* or SYD985*):ti,ab | 2645 |
| 33 | #21 or #28 or #29 or #30 or #31 or #32 | 3379 |
| 34 | #10 and #20 and #33 | 128 |
| 35 | #34 in Trials | 127 |

###

###

### Table S4. Inclusion/exclusion criteria for abstract/title screening

| **1.** **Does this study examine targeted therapy in HER2-positive breast cancer?** | |
| --- | --- |
| **INCLUDE** | **EXCLUDE** |
| - The study, or portion of the study, assesses patient outcomes for HER2-targeted therapy in HER2-positive breast cancer | Studies that ONLY report:   - HER2-negative disease - Other therapeutic modalities |
| **2. Year/language/setting/study length** | |
| **INCLUDE** | **EXCLUDE** |
| - English - Studies from all available years - All settings - No restrictions on the length of study | - Not in English |
| **3.** **Participants** | |
| **INCLUDE** | **EXCLUDE** |
| - Adult human population (age 18 or older) - HER2+ breast cancer | - Non-human studies |
| **4.** **Intervention** | |
| **INCLUDE** | **EXCLUDE** |
| - Studies reporting targeted therapy for HER2-positive breast cancer either as:   - monotherapy OR combination with other treatment modalities   - first-line therapy OR any line of therapy   **List of HER2 targeted therapies**   - Trastuzumab (Herceptin) - Trastuzumab-dkst (Ogivri) - Pertuzumab (Perjeta) - Trastuzumab emtansine (Kadcyla or T-DM1) - Lapatinib (Tykerb) - Neratinib (Nerlynx) - Tucatinib - Ertumaxomab (Rexomun) - Pyrotinib - Epertinib - SYD-985 - DS-8201 | - Articles that do not include HER2-targeted therapy |
| **5.** **Study design** | |
| **INCLUDE** | **EXCLUDE** |
| - Randomized control trial (RCT) - Non-randomized control trial (RCT) - Prospective cohort study - Retrospective cohort study | - Case-control study - Cross-sectional study - Case report - Case series - Review - Additional types:   - Organization reports   - Research thesis (e.g., PhD, Master’s, undergraduate)   - Best practices, use cases   - Protocol   - Theoretical models   - Interviews/Observation   - Editorials, commentaries   - Opinion pieces |

### Table S5. Inclusion/exclusion criteria for full-text review

| 1. **Participants** | |
| --- | --- |
| **INCLUDE** | **EXCLUDE** |
| - Brain metastases from HER2+ breast cancer - Leptomeningeal metastases from HER2+ breast cancer | - Studies that do not involve patients with brain metastases from HER2+ breast cancer - Studies with fewer than 5 patients with brain metastases from HER2+ breast cancer |
| 1. **Outcomes** | |
| **INCLUDE** | **EXCLUDE** |
| - Studies that report any of the following outcomes specific to patients with brain metastases from HER2+ breast cancer:   - Overall survival (OS)   - Progression-free survival (PFS)   - Adverse events (of special interest: adverse neurologic events)   - Progression-free survival, intracranial (iPFS)   - Objective response rate, intracranial (iORR)   - Complete response rate, intracranial (iCRR)   - Partial response rate, intracranial (iPRR)   - Disease control rate, intracranial (iDCR)   - Time to response, intracranial (iTTR)   - Best change in lesion size, intracranial | - Studies that do not report any of the outcomes mentioned - Studies that only report the mentioned outcomes for other patient groups |
| 1. **Separate publications for subgroup analysis** | |
| **INCLUDE** | **EXCLUDE** |
| - In cases where a study of patients with breast cancer publishes a separate subgroup analysis of patients with brain metastases from breast cancer, the subgroup analysis publication will be included, and the main analysis will be excluded. - In cases where a study of patients with breast cancer publishes a separate subgroup analysis of patients with HER2+ disease, the subgroup analysis publication will be included, and the main analysis will be excluded. |  |

###

### Table S6. Median follow-up

| Author | Year | Patients | Therapy | Median follow-up (months) |
| --- | --- | --- | --- | --- |
| Jacot, W. et al. | 2016 | 39 | T-DM1 | 8.1 (range 1.4-39.6) |
| Mailliez, A. et al. | 2016 | 14 | T-DM1 | 12 (95% CI, 5.4-35.9) |
| Bachelot, T. et al. | 2011 | 45 | Lapatinib + capecitabine | 21.2 (range 2.2-27.6) |
| Mounsey, L. et al. | 2018 | 123 | Trastuzumab, lapatinib, T-DM1, and/or pertuzumab | 21 |
| Metro, G. et al. | 2007 | 20 | Trastuzumab | 17 (range 2-57) |
| Metro, G. et al. | 2011 | 53 | Lapatinib + capecitabine | 26 (range 5-71) |
| Le Scodan, R. et al. | 2011 | 52 | Trastuzumab | 6.25 (range 0.23-53) |
| Fabi, A. et al. | 2018 | 87 | T-DM1 | 16 (range 1-55) |
| Kim, J. et al. | 2019 | 84 | Lapatinib + SRS | 8.2 (IQR 3.3-17.5) |
| Kaplan, M. et al. | 2013 | 111 | Lapatinib + capecitabine | Lapatinib: 17, trastuzumab: 19mo |
| Bidard, F. et al. | 2009 | 6 (LM) | Trastuzumab +/- lapatinib | 12.5 (range 2-18) |
| Bhargava, P. et al. | 2019 | 102 | Lapatinib and/or Trastuzumab or T-DM1 or trastuzumab intrathecal | 13.5 |
| Bartsch, R. et al. | 2011 | 80 | Trastuzumab +/- lapatinib | 24 (range 8-46) |
| Zhang, C. et al. | 2016 | 68 | Trastuzumab | Mean±SD: 17.2 ± 9.3 (range 1-39) |
| Pierga, J. et al. | 2013 | 44 | Lapatinib + capecitabine | 21.2 |
| Parsai, S. et al. | 2019 | 126 | Lapatinib + SRS | 17.1 |
| Shawky, H. et al. | 2014 | 21 | Lapatinib + capecitabine | 11.0 (range 1.57-32.57) |
| Yomo, S. et al. | 2013 | 40 | Lapatinib + SRS | 10.3 |
| Okines, A. et al. | 2018 | 16 | T-DM1 | 20.5 |

###

### Table S7. Pharmaceutical industry funding among reporting studies.

| **Author** | **Year** | **Patients (n)** | **Therapy** | **Pharmaceutical funding** |
| --- | --- | --- | --- | --- |
| Krop, I. et al. | 2015 | 95 | T-DM1 | Study |
| Yap, Y. et al. | 2012 | 280 | Trastuzumab and/or lapatinib | Study |
| Brufsky, A. et al. | 2011 | 377 | Trastuzumab | Study |
| Murthy, R. et al. | 2019 | 291 | Tucatinib+trastuzumab+capecitabine | Study |
| Takano, T. et al. | 2018 | 13 | Trastuzumab+capecitabine | Study |
| Morikawa, A. et al. | 2019 | 11 | Lapatinib+capecitabine | Study |
| Martin Huertas, R. et al. | 2019 | 8 | T-DM1 | Study |
| MacPherson, I. et al. | 2019 | 5 | Trastuzumab + epertinib or capecitabine | Study |
| Lin, N. et al. | 2013 | 35 | Lapatinib+WBRT+Trastuzumab | Study |
| Lin, N. et al. | 2011 | 22 | Lapatinib + capecitabine or topotecan | Study |
| Lin, N. et al. | 2009 | 242 | Lapatinib | Study |
| Leone, J. et al. | 2019 | 21 | Trastuzumab+cabozantinib | Study |
| Hurvitz, S. et al. | 2018 | 19 | Lapatinib+everolimus+capecitabine | Study |
| Gutierrez, M. et al. | 2015 | 19 | Trastuzumab (intrathecal) | Study |
| Jackisch, C. et al. | 2014 | 90 | Trastuzumab | Study |
| de Azambuja, E. et al. | 2013 | 16 | Lapatinib+temozolomide | Study |
| Borges, V. et al. | 2018 | 30 | Tucatinib+T-DM1 | Study |
| Bonneau, C. et al. | 2018 | 16 | Trastuzumab (intrathecal) | Study |
| Gavila, J. et al. | 2019 | 38 | Trastuzumab+lapatinib | Study |
| Freedman, R. et al. | 2019 | 40 | Neratinib | Study |
| Van Swearingen, A. et al. | 2018 | 32 | Trastuzumab+everolimus+vinorelbine | Study |
| Bachelot, T. et al. | 2011 | 45 | Lapatinib+capecitabine | Study |
| Christodoulou, C. et al. | 2017 | 12 | Lapatinib+WBRT | Study |
| Sutherland, S. et al. | 2010 | 34 | Lapatinib+capecitabine | Study |
| Ro, J. et al. | 2012 | 58 | Lapatinib+capecitabine | Study |
| Pistilli, B. et al. | 2018 | 9 | Trastuzumab+buparlisib+capecitabine | Study |
| Yardley, D. et al. | 2015 | 26 | T-DM1 | Study |
| Yardley, D. et al. | 2018 | 11 | Lapatinib+cabazitaxel | Study |
| Murthy, R. et al. | 2018 | 29 | Tucatinib +/- capecitabine +/- trastuzumab | Study |
| Parsai, S. et al. | 2019 | 126 | Lapatinib+SRS | Authors |
| Hayashi, N. et al. | 2015 | 432 | Trastuzumab and/or lapatinib | Authors |
| Mounsey, L. et al. | 2018 | 123 | Trastuzumab, lapatinib, T-DM1, and/or pertuzumab | Authors |
| Miller, J. et al. | 2017 | 99 | Trastuzumab or lapatinib or pertuzumab or T-DM1 | Authors |
| Bartsch, R. et al. | 2011 | 80 | Trastuzumab +/- lapatinib | Authors |
| Mueller, V. et al. | 2016 | 472 | Trastuzumab or lapatinib or T-DM1 or trastuzumab+pertuzumab | Authors |
| Witzel, I. et al. | 2011 | 29 | Trastuzumab | Authors |
| Kim, J. et al. | 2019 | 84 | Lapatinib+SRS | Authors |
| Church, D. et al. | 2008 | 26 | Trastuzumab | Authors |
| Montagna, E. et al. | 2009 | 36 | Trastuzumab | Authors |
| Mc, Cabe Y. et al. | 2016 | 23 | T-DM1 | Authors |
| Lin, N. et al. | 2008 | 39 | Lapatinib | Authors |
| Hardy-Werbin, M. et al. | 2019 | 5 | T-DM1 | Authors |
| Gori, S. et al. | 2012 | 16 | Trastuzumab | Authors |
| Falchook, G. et al. | 2013 | 10 | Trastuzumab+lapatinib+bevacizumab | Authors |
| Jacot, W. et al. | 2016 | 39 | T-DM1 | Authors |
| Bhargava, P. et al. | 2019 | 102 | Lapatinib and/or trastuzumab or T-DM1 or trastuzumab (intrathecal) | Authors |
| Vasista, A. et al. | 2019 | 29 | Trastuzumab | Authors |
| Bartsch, R. et al. | 2009 | 40 | Trastuzumab | Authors |
| Griguolo, G. et al. | 2018 | 32 | Pertuzumab, trastuzumab, T-DM1, and/or lapatinib | None |
| Morikawa, A. et al. | 2018 | 100 | Trastuzumab and/or lapatinib | None |
| Zhang, Q. et al. | 2016 | 60 | Trastuzumab and/or lapatinib | None |
| Zhang, C. et al. | 2016 | 68 | Trastuzumab | None |
| Gori, S. et al. | 2019 | 154 | Trastuzumab and/or lapatinib | None |
| Gomes, D. et al. | 2015 | 326 | Trastuzumab and/or lapatinib | None |
| Kaplan, M. et al. | 2015 | 50 | Trastuzumab +/- lapatinib | None |
| Le Scodan, R. et al. | 2011 | 52 | Trastuzumab | None |
| Karam, I. et al. | 2011 | 176 | Trastuzumab+RT | None |
| Yomo, S. et al. | 2013 | 40 | Lapatinib+SRS | None |
| Hulsbergen, A. et al. | 2020 | 15 | Trastuzumab and/or lapatinib | None |
| Metro, G. et al. | 2007 | 20 | Trastuzumab | None |
| Metro, G. et al. | 2011 | 53 | Lapatinib+capecitabine | None |
| Kaplan, M. et al. | 2013 | 111 | Lapatinib+capecitabine | None |
| Braccini, A. et al. | 2013 | 109 | Trastuzumab and/or lapatinib | None |
| Bian, L. et al. | 2013 | 16 | Trastuzumab+capecitabine | None |
| Chen, J. et al. | 2014 | 60 | HER2-targeted therapy | None |
| Ou, Dan. et al. | 2019 | 39 | HER2-targeted therapy | None |
| Park, I. et al. | 2009 | 78 | Trastuzumab | None |
| Okita, Y. et al. | 2013 | 27 | Trastuzumab | None |
| Niwinska, A. et al. | 2010 | 223 | trastuzumab and/or lapatinib | None |
| Metro, G. et al. | 2010 | 10 | Trastuzumab + chemotherapy or endocrine therapy | None |
| Mailliez, A. et al. | 2016 | 14 | T-DM1 | None |
| Naskhletashvili, D. et al. | 2010 | 5 | Trastuzumab+capecitabine | None |
| Huang, C. et al. | 2010 | 52 | Lapatinib+capecitabine | None |
| Grell, P. et al. | 2012 | 31 | Lapatinib | None |
| Giotta, F. et al. | 2010 | 14 | Lapatinib+capecitabine | None |
| Figura, N. et al. | 2019 | 18 | Trastuzumab (intrathecal) | None |
| Fabi, A. et al. | 2018 | 87 | T-DM1 | None |
| Vici, P. et al. | 2017 | 61 | T-DM1 | None |
| Gamucci, T. et al. | 2019 | 21 | Pertuzumab+trastuzumab+taxanes | None |
| Toi, M. et al. | 2009 | 10 | Lapatinib | None |
| Rossi, M. et al. | 2016 | 40 | Trastuzumab | None |
| Riahi, H. et al. | 2010 | 31 | Trastuzumab+WBRT | None |
| Shawky, H. et al. | 2014 | 21 | Lapatinib+capecitabine | None |
| Niwinska, A. et al. | 2010 | 52 | Trastuzumab+chemotherapy | None |

###

### Table S8. Summary estimates for individual HER2-targeted agents.

Studies were allowed to contribute to summary estimates for individual HER2-targeted agents if their treatment arm involved any receipt of that agent, and the comparator arm did not. For example, a study reporting a hazard ratio for overall survival in patients who received “trastuzumab, lapatinib, and/or T-DM1” vs. “non-targeted therapy” was included in the meta-analyses for each of trastuzumab, lapatinib, and T-DM1 for overall survival.

| **Overall survival (OS)** | **HR** | **95% CI** | **n** | **k** | ***I*^2^** |
| --- | --- | --- | --- | --- | --- |
| Trastuzumab | 0.45 | 0.37–0.54 | 2567 | 19 | 63% |
| Lapatinib | 0.46 | 0.36–0.58 | 1591 | 14 | 62% |
| Pertuzumab | 0.64 | 0.49–0.84 | 254 | 3 | 47% |
| Tucatinib | 0.58 | 0.40–0.85 | 291 | 1 | N/A |
| T-DM1 | 0.64 | 0.49–0.84 | 254 | 3 | 47% |
| **Progression-free survival (PFS)** | **HR** | **95% CI** | **n** | **k** | ***I*^2^** |
| Trastuzumab | 0.52 | 0.27–1.02 | 475 | 4 | 79% |
| Lapatinib | 0.26 | 0.05–1.34 | 15 | 1 | N/A |
| Tucatinib | 0.46 | 0.31–0.68 | 291 | 1 | N/A |
| **Intracranial objective response rate (iORR)** | **Rate** | **95% CI** | **n** | **k** | ***I*^2^** |
| Trastuzumab | 19% | 6–36% | 257 | 14 | 89% |
| Lapatinib | 22% | 12–33% | 613 | 18 | 88% |
| Neratinib | 8% | 1–18% | 40 | 1 | N/A |
| Pertuzumab | 11% | 2–23% | 37 | 1 | N/A |
| Tucatinib | 25% | 5–51% | 62 | 3 | 76% |
| T-DM1 | 23% | 8–40% | 104 | 5 | 62% |
| **Intracranial disease control rate (iDCR)** | **Rate** | **95% CI** | **n** | **k** | ***I*^2^** |
| Trastuzumab | 66% | 54–76% | 202 | 12 | 49% |
| Lapatinib | 63% | 52–73% | 615 | 18 | 80% |
| Neratinib | 48% | 33–63% | 40 | 1 | N/A |
| Pertuzumab | 48% | 36–61% | 58 | 2 | 0% |
| Tucatinib | 84% | 71–92% | 50 | 2 | 0% |
| T-DM1 | 56% | 46–65% | 103 | 5 | 28% |
| **Intracranial complete response rate (iCRR)** | **Rate** | **95% CI** | **n** | **k** | ***I*^2^** |
| Trastuzumab | 0% | 0–4% | 225 | 12 | 39% |
| Lapatinib | 0% | 0–2% | 571 | 15 | 31% |
| Neratinib | 0% | 0–4% | 40 | 1 | N/A |
| Pertuzumab | 0% | 0–5% | 37 | 1 | N/A |
| Tucatinib | 2% | 0–13% | 62 | 3 | 58% |
| T-DM1 | 4% | 0–12% | 83 | 3 | 30% |
| **Grade 3+ adverse event rate** | **Rate** | **95% CI** | **n** | **k** | ***I*^2^** |
| Trastuzumab | 0% | 0–7% | 53 | 3 | 26% |
| Lapatinib | 58% | 50–67% | 144 | 4 | 0% |
| T-DM1 | 21% | 5–45% | 495 | 4 | 90% |

###

### Table S9. Overall survival (OS) as reported in months

| Author | Year | Patients | Therapy | Comparative | Median OS (months) |
| --- | --- | --- | --- | --- | --- |
| Mueller, V. et al. | 2016 | 472 | Trastuzumab or lapatinib or T-DM1 or trastuzumab+pertuzumab | Yes | Anti-HER2 vs control: 17.7 (95% CI, 15-22) vs 12.9 (95% CI, 10.5-17.1) , p = 0.015 |
| Mounsey, L. et al. | 2018 | 123 | Trastuzumab, lapatinib, T-DM1, and/or pertuzumab | Yes | Anti-HER2 vs control: 2.11yr (95% CI, 1.55-2.60) vs 0.65yr (95% CI, 0.38-1.25, p = .001) |
| Morikawa, A. et al. | 2018 | 100 | Trastuzumab and/or lapatinib | Yes | Anti-HER2 (with lapatinib) vs anti-HER2 (without lapatinib) vs control: 31.8 (95% CI, 19.6-45.5) vs 20 (95% CI, 12-26.8) vs 7.2 (95% CI, 3.5-16.2), p<0.001 |
| Miller, J. et al. | 2017 | 99 | Trastuzumab or lapatinib or pertuzumab or T-DM1 | Yes | Anti-HER2 antibody vs control: 17.9 vs 15.1mo (p = .04), HR 0.70 (95% CI, 0.49-0.98); HER2/EGFR TKI vs control: 21.1mo vs 15.4mo (p = .03), HR 0.79 (95% CI, 0.58-1.08) |
| Metro, G. et al. | 2007 | 20 | Trastuzumab | Yes | Trastuzumab vs chemotherapy: Not reached (cutoff at 36mo) vs 11 (95% CI, 1-22), p = .008 |
| Metro, G. et al. | 2011 | 53 | Lapatinib+capecitabine | Yes | Lapatinib + capecitabine vs trastuzumab: 27.9 vs 16.7, p = 0.01 |
| Witzel, I. et al. | 2011 | 29 | Trastuzumab | Yes | Trastuzumab vs no trastuzumab: 9 (95% CI, 7-11) vs 2 (95% CI, 0.7‚-3), p = 0.006 |
| Le Scodan, R. et al. | 2011 | 52 | Trastuzumab | Yes | Trastuzumab vs no trastuzumab: 19.53 (95% CI, 9.27-not reached) vs 5.6 (95% CI, 2.60-12.49) |
| Hulsbergen, A. et al. | 2020 | 15 | Trastuzumab and/or lapatinib | Yes | HER2-targeted therapy vs no HER2-targeted therapy: 22 vs 43.7 |
| Hayashi, N. et al. | 2015 | 432 | Trastuzumab and/or lapatinib | Yes | Lapatinib vs no lapatinib: HR 0.510 (95% CI, 0.383-0.679); trastuzumab vs no trastuzumab: HR 0.445 (95% CI, 0.352-0.563) |
| Griguolo, G. et al. | 2018 | 32 | Pertuzumab, trastuzumab, T-DM1, and/or lapatinib | Yes | HER2-targeted therapy vs not: 11.4 (95% CI; 4.5-18.4) vs 1.7 (95% CI, 0.6-2.8) , p<0.001 |
| Gori, S. et al. | 2019 | 154 | Trastuzumab and/or lapatinib | Yes | HER2-targeted therapy vs no HER2-targeted therapy: 27.5 vs 13.8 |
| Kim, J. et al. | 2019 | 84 | Lapatinib+SRS | Yes | Lapatinib vs no lapatinib: 33.3 vs 23.6 (p = 0.009) |
| Kaplan, M. et al. | 2013 | 111 | Lapatinib+capecitabine | Yes | Lapatinib vs trastuzumab: 19.1 vs 12 , p = 0.039 |
| Kaplan, M. et al. | 2015 | 50 | Trastuzumab +/- Lapatinib | Yes | Trastuzumab vs no trastuzumab: 11.4 vs 4.1mo (p = .012); OS for lapatinib vs no lapatinib: 15.9mo vs 5.6mo (p = .01), HR: 0.33 (0.14-0.80) |
| Braccini, A. et al. | 2013 | 109 | Trastuzumab and/or lapatinib | Yes | HER2-targeted therapy vs no HER2-targeted therapy: 15.2 (95% CI, 11.5-19.4) vs 3.4 (95% CI, 1.4-6), (p < 0.001); lapatinib+trastuzumab vs lapatinib or trastuzumab: 25.7mo (95% CI, 17.1-33.3) vs 9.6mo (95% CI, 8.2‚-12.8mo) |
| Krop, I. et al. | 2015 | 95 | T-DM1 | Yes | T-DM1 vs lapatinib+capecitabine: 26.8 vs 12.9mo; HR 0.28, no confidence intervals reported. |
| Bartsch, R. et al. | 2007 | 53 | Trastuzumab | Yes | trastuzumab vs chetherapy: 21mo (95% CI, 11.28-30.72) vs 9mo (95% CI, 2.06‚-14.99), p <0.001 |
| Church, D. et al. | 2008 | 26 | Trastuzumab | Yes | trastuzumab vs no trastuzumab: 11.9 vs 3.0mo (p = .05) |
| Chen, J. et al. | 2014 | 60 | HER2-targeted therapy | Yes | HER2-targeted therapy vs no HER2-targeted therapy: 21 vs 9mo (p = .002) |
| Brufsky, A. et al. | 2011 | 377 | Trastuzumab | Yes | trastuzumab: 17.5 (n=258) vs no trastuzumab: 3.7mo (n=119) |
| Ou, Dan. et al. | 2019 | 39 | HER2-targeted therapy | Yes | HER2-targeted therapy vs no HER2-targeted therapy: 24.5 vs 5.6mo (p = .001) |
| Tarhan, M. et al. | 2013 | 36 | Trastuzumab and/or lapatinib | Yes | HER2-targeted therapy+chemotherapy vs chemotherapy: 17 (95% CI, 11.9-22) vs 11.5 (95% CI, 6.35‚-16.6) |
| Zhukova, L. et al. | 2018 | 64 | Trastuzumab +/- lapatinib | Yes | HER2-targeted therapy vs never HER2-targeted therapy vs no HER2-targeted therapy after diagnosis of brain metastasis: 25.3 vs 5.24 vs 2.1 (p = .001) |
| Zhang, Q. et al. | 2016 | 60 | Trastuzumab and/or lapatinib | Yes | HER2-targeted therapy without adjuvant HER2-targeted therapy vs HER2-targeted therapy with adjuvant HER2-targeted therapy vs never HER2-targeted therapy: 27 (95% CI, 10.97-43.03) vs 16 (95% CI, 4.87‚-27.13) vs 9 (95% CI, 6.24‚-11.76). Any HER2-targeted therapy post-brain metastasis: 21. |
| Zhang, C. et al. | 2016 | 68 | Trastuzumab | Yes | Trastuzumab vs no trastuzumab: 21.2 (± 9.2) versus 13.5 (± 7.7) (Mean ± SD) |
| Parsai, S. et al. | 2019 | 126 | Lapatinib+SRS | Yes | Lapatinib vs no lapatinib: 27.3 vs 19.5 (p = .03) |
| Park, Y. et al. | 2009 | 77 | Trastuzumab | Yes | Trastuzumab vs no trastuzumab, HR: 0.278 (95% CI, 0.064-1.199); 14.9 (95% CI 11.6-18.2) vs 4.0 (95% CI, 2.1-5.9); lapatinib vs no lapatinib, HR: 0.197 (95% CI, 0.042-0.926) |
| Park, I. et al. | 2009 | 78 | Trastuzumab | Yes | Trastuzumab vs no trastuzumab: 13.6 (95% CI, 9.0-18.2) vs 5.5 (95% CI, 0.0-13.6) |
| Yomo, S. et al. | 2013 | 40 | Lapatinib+SRS | Yes | Lapatinib vs no lapatinib: 19.5 (95% CI, 11.0-not reached) vs 15.0 (95% CI, 9.3-28.1) |
| Okita, Y. et al. | 2013 | 27 | Trastuzumab | Yes | Trastuzumab vs no trastuzumab: 38 vs 8.4 (p = .0005) |
| Niwinska, A. et al. | 2010 | 223 | Trastuzumab and/or lapatinib | Yes | Luminal B, HER2-targeted therapy vs chemotherapy vs no systemic treatment: 15 (95% CI, 10.08-19.80) vs 9 (95% CI, 6.60-11.52) vs 2 (95% CI, 2.04-2.76); HER2-overexpressing, HER2-targeted therapy vs chemotherapy vs no systemic treatment: 13 (95% CI, 9.96-16.44) vs 6 (95% CI, 4.56-7.92) vs 4 (95% CI, 3.36-4.32) |
| Niwinska, A. et al. | 2013 | 303 | Trastuzumab or lapatinib | Yes | Trastuzumab monotherapy vs trastuzumab+chemotherapy vs lapatinib+chemotherapy vs trastuzumab+lapatinib+chemotherapy: 8 vs 15 vs 20 vs 29 (p = .233) |
| Yap, Y. et al. | 2012 | 280 | Trastuzumab and/or lapatinib | Yes | HER2-targeted therapy vs no HER2-targeted therapy: 18.5 (95% CI, 12.9-21.8) vs 5.7 (95% CI, 4.2‚-8.9); lapatinib+trastuzumab vs lapatinib vs trastuzumab: 25.9 vs 21.4 vs 10.5 (p<.001) |
| Bonneau, C. et al. | 2018 | 16 | Trastuzumab (intrathecal) | No | 7.3 (range 0.4-27.9) |
| Riahi, H. et al. | 2010 | 31 | Trastuzumab+WBRT | No | 18 (range 2-65) |
| Jacot, W. et al. | 2016 | 39 | T-DM1 | No | Not reached (1-year OS 58%; 95% CI, 10-75%) |
| Mailliez, A. et al. | 2016 | 14 | T-DM1 | No | 9.07 (95% CI, 3.65-24.8) |
| Bachelot, T. et al. | 2011 | 45 | Lapatinib+capecitabine | No | 17.0 (95% CI, 13.7-24.9) |
| Montagna, E. et al. | 2009 | 36 | Trastuzumab | No | 25.4 (95% CI, 15.2-not reached) |
| Metro, G. et al. | 2010 | 10 | Trastuzumab+chemotherapy or ET | No | 13 |
| Mc, Cabe Y. et al. | 2016 | 23 | T-DM1 | No | 85 weeks, 95% CI 81-107 |
| Lin, N. et al. | 2013 | 35 | Lapatinib+WBRT+trastuzumab | No | 19 (range 1-62) |
| Lin, N. et al. | 2009 | 242 | Lapatinib | No | 6.37 (95% CI, 5.49-8.25) |
| Leone, J. et al. | 2019 | 21 | Trastuzumab+cabozantinib | No | 13.8 (95% CI; 8.2-not reached) |
| Hurvitz, S. et al. | 2018 | 19 | Lapatinib+everolimus+capecitabine | No | 24.2 (95% CI, 6.2-25.4) |
| Hardy-Werbin, M. et al. | 2019 | 5 | T-DM1 | No | Not reached |
| Grell, P. et al. | 2012 | 31 | Lapatinib | No | Not reached. 67.3% at 6 mo |
| Gori, S. et al. | 2012 | 16 | Trastuzumab | No | 17.3 (95% CI 3.4-32.2) |
| Figura, N. et al. | 2019 | 18 | Trastuzumab (intrathecal) | No | 13.2 (95% CI 4.4-not reached) |
| Fabi, A. et al. | 2018 | 87 | T-DM1 | No | 14 (95% CI, 12.2-15.8) |
| Jackisch, C. et al. | 2014 | 90 | Trastuzumab | No | 20.3 |
| Vici, P. et al. | 2017 | 61 | T-DM1 | No | 16 (95% CI, 12-19) |
| de Azambuja, E. et al. | 2013 | 16 | Lapatinib+temozolomide | No | 10.94 (95% CI, 1.09-20.79) |
| Bartsch, R. et al. | 2009 | 40 | Trastuzumab | No | 10 (95% CI, 4.53-15.47) |
| Bidard, F. et al. | 2009 | 6 (LM) | Trastuzumab +/- lapatinib | No | 12.5 (range 2-18) |
| Bhargava, P. et al. | 2019 | 102 | Lapatinib and/or trastuzumab or T-DM1 or trastuzumab (intrathecal) | No | 14 (95% CI, 10.8-17.2), 2-year OS 25% (95% CI, 16.7-34.4%) |
| Gavila, J. et al. | 2019 | 38 | Trastuzumab+lapatinib | No | 15.2 |
| Gamucci, T. et al. | 2019 | 21 | Pertuzumab+trastuzumab+taxanes | No | 2-year OS 77.7% |
| Vasista, A. et al. | 2019 | 29 | Trastuzumab | No | 21 (IQR 11-29) |
| Freedman, R. et al. | 2019 | 40 | Neratinib | No | 8.7, 1-year OS 30% |
| Van Swearingen, A. et al. | 2018 | 32 | Trastuzumab+everolimus+vinorelbine | No | 12.12 (95% CI, 6.84-21.36) |
| Bartsch, R. et al. | 2011 | 80 | Trastuzumab +/- lapatinib | No | 18 (95% CI, 12.49-23.51) |
| Christodoulou, C. et al. | 2017 | 12 | Lapatinib+WBRT | No | 17 (95% CI, 8.1-25.3mo) |
| Chmielowska, E. et al. | 2017 | 2 | Lapatinib+capecitabine | No | 11.5 |
| Chan, A. et al. | 2019 | 101 | AC-TH or TCH | No | AC-T: 42.5 (28.3-62.7), AC-TH: 53.2 (31.2-103.6), TCH: 30.3 (23.4-39.0) |
| Rossi, M. et al. | 2016 | 40 | Trastuzumab | No | 1-year OS, 47.5% |
| Ro, J. et al. | 2012 | 58 | Lapatinib+capecitabine | No | 11.3 |
| Shawky, H. et al. | 2014 | 21 | Lapatinib+capecitabine | No | 11; 6-month OS, 80.6%; 1-year OS, 45.6% |
| Okines, A. et al. | 2018 | 16 | T-DM1 | No | 15.3 (95% CI, 4.7-not reached) |
| Niwinska, A. et al. | 2010 | 52 | Trastuzumab+chemotherapy | No | 8.78 (range 0.5-32) |

###

### Table S10. Progression-free survival (PFS) as reported in months

| Author | Year | Patients | Therapy | Comparative | Median PFS (months) |
| --- | --- | --- | --- | --- | --- |
| Metro, G. et al. | 2011 | 53 | Lapatinib + capecitabine | Yes | 5.1 (95% CI, 2.6-7.5) |
| Hulsbergen, A. et al. | 2020 | 15 | Trastuzumab and/or lapatinib | Yes | HER2-targeted therapy vs no HER2-targeted therapy: 7.1 vs 3.3 |
| Krop, I. et al. | 2015 | 95 | T-DM1 | Yes | T-DM1 vs lapatinib+capecitabine: 5.9 vs 5.7 |
| Chan, A. et al. | 2019 | 101 | AC-TH or TCH | Yes | AC-T vs AC-TH vs TCH: 23.8 (95% CI, 13.3-30.4) vs 19.9 (95% CI, 16.6-25.1) vs 19.9 (95% CI, 15.0-27.2) |
| Zhang, C. et al. | 2016 | 68 | trastuzumab | Yes | Trastuzumab vs. no trastuzumab: 9.0 (± 4.6) vs 5.2 (± 4.1) (Mean ± SD) |
| Murthy, R. et al. | 2019 | 291 | Tucatinib+trastuzumab+capecitabine | Yes | Tucatinib vs placebo: 7.6 (95% CI, 6.2-9.5) vs 5.4 (95% CI, 4.1-5.7) |
| Jacot, W. et al. | 2016 | 39 | T-DM1 | No | 6.1 (95% CI, 5.2-18.3) |
| Lin, N. et al. | 2008 | 39 | Lapatinib | No | PFS 18% at 16 weeks |
| Montemurro, F. et al. | 2017 | 399 | T-DM1 | No | 5.5 |
| Martin Huertas, R. et al. | 2019 | 8 | T-DM1 | No | 5 (95% CI, 3.88-6.50) |
| Lin, N. et al. | 2013 | 35 | Lapatinib+WBRT+trastuzumab | No | 4.8 (range 0-58.3) |
| Lin, N. et al. | 2009 | 242 | Lapatinib | No | 2.40 (95% CI, 1.87-2.79) |
| Leone, J. et al. | 2019 | 21 | Trastuzumab+cabozantinib | No | 4.1 (95% CI, 2.8 - 6.2) |
| Hurvitz, S. et al. | 2018 | 19 | Lapatinib+everolimus+capecitabine | No | 6.2 (95% CI, 3.2-9.1) |
| Huang, C. et al. | 2010 | 52 | Lapatinib+capecitabine | No | 8.47 |
| Hardy-Werbin, M. et al. | 2019 | 5 | T-DM1 | No | 6 (95% CI, 3.85-8.14) |
| Grell, P. et al. | 2012 | 31 | Lapatinib | No | 6.2 (95% CI; 3.3-9.1) |
| Figura, N. et al. | 2019 | 18 | Trastuzumab (intrathecal) | No | 7.2 ( 95% CI 2.7-25.7) |
| Fabi, A. et al. | 2018 | 87 | T-DM1 | No | 7 (95% CI: 5.4-8.6) |
| Jackisch, C. et al. | 2014 | 90 | Trastuzumab | No | 7.5 |
| Vici, P. et al. | 2017 | 61 | T-DM1 | No | 6 (95% CI, 4-8) |
| de Azambuja, E. et al. | 2013 | 16 | Lapatinib+temozolomide | No | 2.60 (95% CI, 1.82-3.37) |
| Borges, V. et al. | 2018 | 30 | Tucatinib+T-DM1 | No | 6.7 (95% CI, 4.1-10.2) |
| Bhargava, P. et al. | 2019 | 102 | Lapatinib and/or Trastuzumab or T-DM1 or trastuzumab (intrathecal) | No | Any HER2-targeted therapy: 8 (95% CI, 6.2-9.8); lapatinib+capecitabine: 9.0 (95% CI, 7.3-10.7) |
| Gamucci, T. et al. | 2019 | 21 | Pertuzumab+trastuzumab+taxanes | No | 20 (95% CI, 13-27), 70.8% at 1 year |
| Ro, J. et al. | 2012 | 58 | Lapatinib+capecitabine | No | 4.3 |
| Shawky, H. et al. | 2014 | 21 | Lapatinib+capecitabine | No | 5.5 (range 1.1-22.0) |
| Yardley, D. et al. | 2015 | 26 | T-DM1 | No | 6.9 (95% CI, 2.7-12.3) |
| Murthy, R. et al. | 2018 | 29 | Tucatinib +/- capecitabine +/- trastuzumab | No | 6.7 (95% CI, 1.4-12.5) |

###

### Table S11. Intracranial progression-free survival (iPFS) as reported in months

| Author | Year | Patients | Therapy | Comparative | Median iPFS (months) |
| --- | --- | --- | --- | --- | --- |
| Gori, S. et al. | 2019 | 154 | Trastuzumab and/or lapatinib | Yes | Trastuzumab vs. other HER2-targeted therapy vs. no HER2-targeted therapy: 10.4 vs. 9.8 vs. 3.5; trastuzumab vs. no HER2-targeted therapy: HR 0.41 (95% CI, 0.27-0.64); other HER2-targeted therapy vs. no HER2-targeted therapy: HR 0.42 (95% CI, 0.27-0.67) |
| Braccini, A. et al. | 2013 | 109 | Trastuzumab and/or lapatinib | Yes | HER2-targeted therapy vs no HER2-targeted therapy: 6.3 (95% CI, 7.8-11.5) vs 5.5 (95% CI, 1.2-6.7), p < 0.001 |
| Metro, G. et al. | 2011 | 53 | Lapatinib+capecitabine | No | 5.6 |
| Mailliez, A. et al. | 2016 | 14 | T-DM1 | No | 2.43 |
| Jacot, W. et al. | 2016 | 39 | T-DM1 | No | 8.6 |
| Figura, N. et al. | 2019 | 18 | Trastuzumab (intrathecal) | No | 5.4 |
| Ro, J. et al. | 2012 | 58 | Lapatinib+capecitabine | No | 7 |

###

### Table S12. Time to intracranial progression (iTTP) as reported in months

| Author | Year | Patients | Therapy | Comparative | Median iTTP (months) |
| --- | --- | --- | --- | --- | --- |
| Metro, G. et al. | 2007 | 20 | Trastuzumab | Yes | Trastuzumab vs chemotherapy: 7 (95% CI, 1-15) vs 10 months (95% CI, 1-21) (p = .48) |
| Gomes, D. et al. | 2015 | 326 | Trastuzumab and/or lapatinib | Yes | Trastuzumab and/or lapatinib vs. no HER2-targeted therapy: HR 0.41 (95% CI, 0.23-0.72) |
| Bartsch, R. et al. | 2007 | 53 | Trastuzumab | Yes | Trastuzumab vs chemotherapy: 9 (95% CI, 4.35-13.65) vs 6 (95% CI, 4.46-7.54) |
| Park, I. et al. | 2009 | 78 | Trastuzumab | Yes | Trastuzumab vs never trastuzumab vs trastuzumab before BM: 7.8 vs. 3.9 vs. 2.9 (p = .006) |
| Riahi, H. et al. | 2010 | 31 | Trastuzumab+WBRT | No | 10.5 (range 2-27) |
| Bachelot, T. et al. | 2011 | 45 | Lapatinib+capecitabine | No | 5.5 (95% CI, 4.5-6.1) |
| Van Swearingen, A. et al. | 2018 | 32 | Trastuzumab+everolimus+vinorelbine | No | 3.9 (95% CI, 2.2-5) |
| Okines, A. et al. | 2018 | 16 | T-DM1 | No | 9.9 (95% CI, 3.9-12.2) |

###

###

### Table S13. Time to progression (TTP) as reported in months

| Author | Year | Patients | Therapy | Comparative | Median TTP (months) |
| --- | --- | --- | --- | --- | --- |
| Lin, N. et al. | 2008 | 39 | Lapatinib | No | 3.0 (95% CI, 2.3 to 3.7) |
| Freedman, R. et al. | 2019 | 40 | Neratinib | No | 1.9 |
| Christodoulou, C. et al. | 2017 | 12 | Lapatinib+WBRT | No | 9.1 (95% CI, 1.7-22.3) |
| Sutherland, S. et al. | 2010 | 34 | Lapatinib+capecitabine | No | 5.5 (95% CI, 3.8-7.0) |

###

### Table S14. Intracranial duration of response (iDoR) as reported in months

| Author | Year | Patients | Therapy | iDoR (months) |
| --- | --- | --- | --- | --- |
| Yardley, D. et al. | 2018 | 11 | Lapatinib+Cabazitaxel | 1.4 |
| Freedman, R. et al. | 2019 | 40 | Neratinib | 3.7 |
| Lin, N. et al. | 2016 | 40 | Pertuzumab+Trastuzumab | 4.6 |
| Metro, G. et al. (Lapatinib arm) | 2011 | 30 | Lapatinib+Capecitabine | 6 (range 3–25) |

###

### Table S15. Frequency of Grade 3+ CTCAE adverse events (AEs), reported as numbers of events

| Author | Year | Patients | Therapy | AEs (n/pts) |
| --- | --- | --- | --- | --- |
| Pistilli, B. et al. | 2018 | 9 | Trastuzumab+Buparlisib+Capecitabine | 2/9 |
| Bonneau, C. et al. | 2018 | 16 | Trastuzumab (intrathecal) | 0/13 |
| Gutierrez, M. et al. | 2015 | 19 | Trastuzumab (intrathecal) | 0/16 |
| Riahi, H. et al. | 2010 | 31 | Trastuzumab+WBRT | 0/31 |
| Shawky, H. et al. | 2014 | 21 | Lapatinib+Capecitabine | 11/21 |
| Metzger, O. et al. | 2017 | 41 | Tucatinib+Trastuzumab | 11/41 |
| Hurvitz, S. et al. | 2018 | 19 | Lapatinib+Everolimus+Capecitabine | 12/19 |
| Freedman, R. et al. | 2019 | 40 | Neratinib | 17/40 |
| Mailliez, A. et al. | 2016 | 14 | T-DM1 | 2/14 |
| Bachelot, T. et al. | 2011 | 45 | Lapatinib+Capecitabine | 31/45 |
| Lin, N. et al. | 2013 | 35 | Lapatinib+WBRT+Trastuzumab | 37/35 |
| Van Swearingen, A. et al. | 2018 | 32 | Trastuzumab+Everolimus+Vinorelbine | 48/32 |
| Morikawa, A. et al. | 2019 | 11 | Lapatinib+Capecitabine | 5/11 |
| Yardley, D. et al. | 2018 | 11 | Lapatinib+Cabazitaxel | 6/11 |
| Kaplan, M. et al. (Lapatinib arm) | 2013 | 46 | Lapatinib+Capecitabine | 11/46 |
| Kaplan, M. et al. (Trastuzumab arm) | 2013 | 65 | Trastuzumab-based therapy | 13/65 |

###

### Table S16. Results of meta-regression analyses for 3-variable models including drug structure, publication year, and study design.

| **Overall survival (k = 24)** | **Beta** | **95% CI** | **Z-value** | **p-value** |
| --- | --- | --- | --- | --- |
| Intercept | 28.9 | -135.38 to 193.17 | 0.34 | 0.73 |
| Structure: Small-molecule inhibitor (Ref: Monoclonal antibody) | 0.01 | -0.53 to 0.55 | 0.04 | 0.97 |
| Year (Range 2009–2020) | -0.01 | -0.1 to 0.07 | -0.36 | 0.72 |
| Study design: RCT (Ref: Prospective cohort study) | 0.79 | -0.31 to 1.88 | 1.41 | 0.16 |
| Study design: Retrospective cohort study (Ref: Prospective cohort study) | 0.38 | -0.48 to 1.24 | 0.86 | 0.39 |
| **Intracranial response rate (k = 36)** | **Beta** | **95% CI** | **Z-value** | **p-value** |
| Intercept | 10.92 | -37.98 to 59.81 | 0.44 | 0.66 |
| Structure: Small-molecule inhibitor (Ref: Monoclonal antibody) | 0.09 | -0.11 to 0.3 | 0.88 | 0.38 |
| Year | -0.01 | -0.03 to 0.02 | -0.42 | 0.68 |
| Study design: Single-arm interventional trial (Ref: Retrospective cohort study) | -0.13 | -0.36 to 0.1 | -1.08 | 0.28 |
| **Intracranial disease control rate (k = 34*)** | **Beta** | **95% CI** | **Z-value** | **p-value** |
| Intercept | -74.76 | -262.32 to 112.79 | -0.78 | 0.43 |
| Structure: Small-molecule inhibitor (Ref: Monoclonal antibody) | 0.44 | -0.36 to 1.24 | 1.08 | 0.28 |
| Year (Range 2008–2019) | 0.04 | -0.06 to 0.13 | 0.78 | 0.44 |
| Study design: Retrospective cohort study (Ref: NRCT) | 0.28 | -1.33 to 1.89 | 0.35 | 0.73 |
| Study design: Single-arm interventional trial (Ref: NRCT) | 0.49 | -1.02 to 1.99 | 0.63 | 0.53 |
| **Intracranial complete response rate (k = 30)** | **Beta** | **95% CI** | **Z-value** | **p-value** |
| Intercept | 1.82 | -22.31 to 25.95 | 0.15 | 0.88 |
| Structure: Small-molecule inhibitor (Ref: Monoclonal antibody) | -0.02 | -0.13 to 0.09 | -0.29 | 0.77 |
| Year (Range 2008–2019) | 0 | -0.01 to 0.01 | -0.13 | 0.9 |
| Study design: Single-arm interventional trial (Ref: Retrospective cohort study) | -0.1 | -0.21 to 0.02 | -1.68 | 0.09 |
| **Grade 3+ adverse event rate (k = 11*)** | **Beta** | **95% CI** | **Z-value** | **p-value** |
| Intercept | 12.47 | -62.19 to 87.13 | 0.33 | 0.74 |
| Structure: Small-molecule inhibitor (Ref: Monoclonal antibody) | 0.33 | 0.04 to 0.61 | 2.26 | 0.02 |
| Year | -0.01 | -0.04 to 0.03 | -0.31 | 0.76 |
| Study design: Retrospective cohort study (Ref: RCT) | -0.47 | -0.81 to -0.13 | -2.69 | 0.01 |
| Study design: Single-arm interventional trial (Ref: RCT) | -0.22 | -0.49 to 0.04 | -1.64 | 0.1 |

*These studies contain k-1 unique studies, but a total of k evaluated study arms.

## Supplementary Figure

**Figure S1. Progression-free survival in patients who received HER2-targeted therapy versus non-targeted therapy.** Hazard ratios for progression-free survival were extracted from eligible studies and pooled in meta-analysis. Studies here are stratified by study design. The size of each box represents the weight of each study in the meta-analysis. The vertical solid line represents the point of equivalence between HER2-targeted therapy and comparators. The vertical dashed and dotted lines represent the points of summary for fixed and random effects models, respectively, and the diamonds represent 95% CI for the summary hazard ratios. Analyses were performed with the R programming language^11^ and the R package meta.^2^


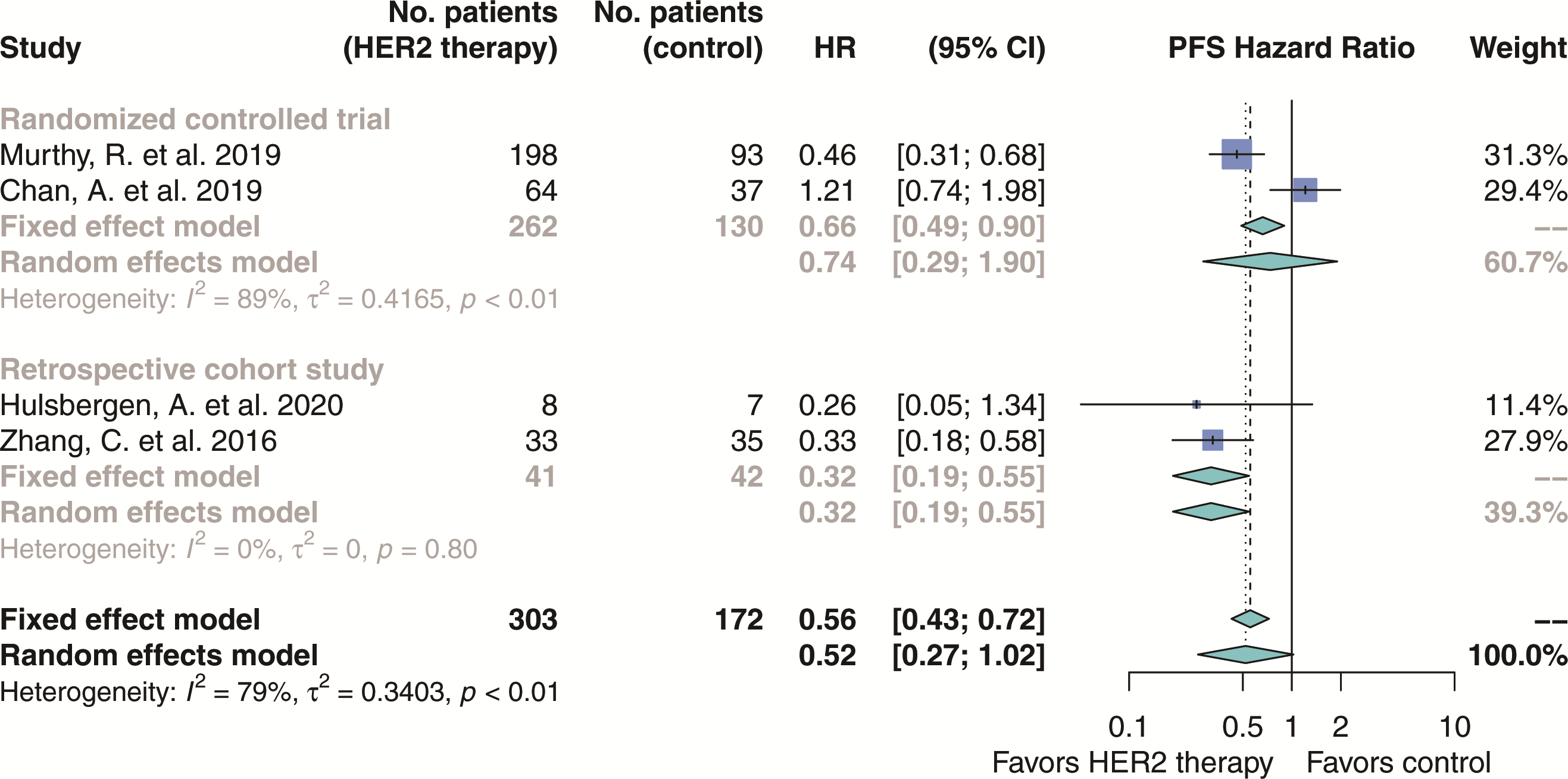


**Figure S2. Intracranial disease control rate in patients who received HER2-targeted therapy.** Proportions for iDCR were extracted from eligible studies and pooled in meta-analysis. Studies here are stratified by study design. The size of each box represents the weight of each study in the meta-analysis. The vertical dashed and dotted lines represent the points of summary for fixed and random effects models, respectively, and the diamonds represent 95% CI for the summary proportions. Analyses were performed with the R programming language^11^ and the R package meta.^2^


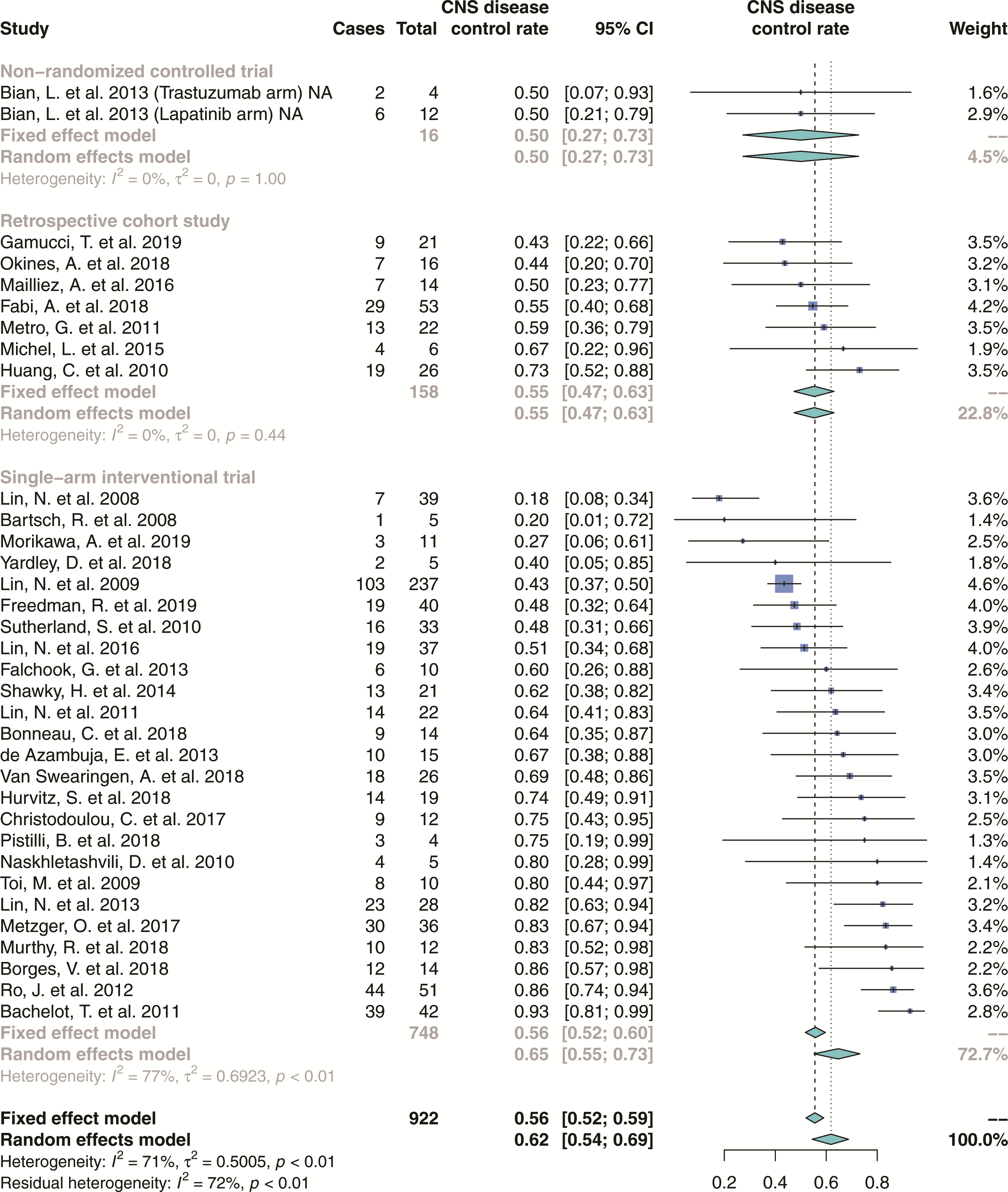


**Figure S3. Intracranial disease control rate in patients who received HER2-targeted therapy, stratified by agent.** Proportions for iDCR were extracted from eligible studies and pooled in meta-analysis. Studies here are stratified by HER2-targeted agent as a subgroup analysis. The size of each box represents the weight of each study in the meta-analysis. The vertical dashed and dotted lines represent the points of summary for fixed and random effects models, respectively, and the diamonds represent 95% CI for the summary proportions. Analyses were performed with the R programming language^11^ and the R package meta.^2^


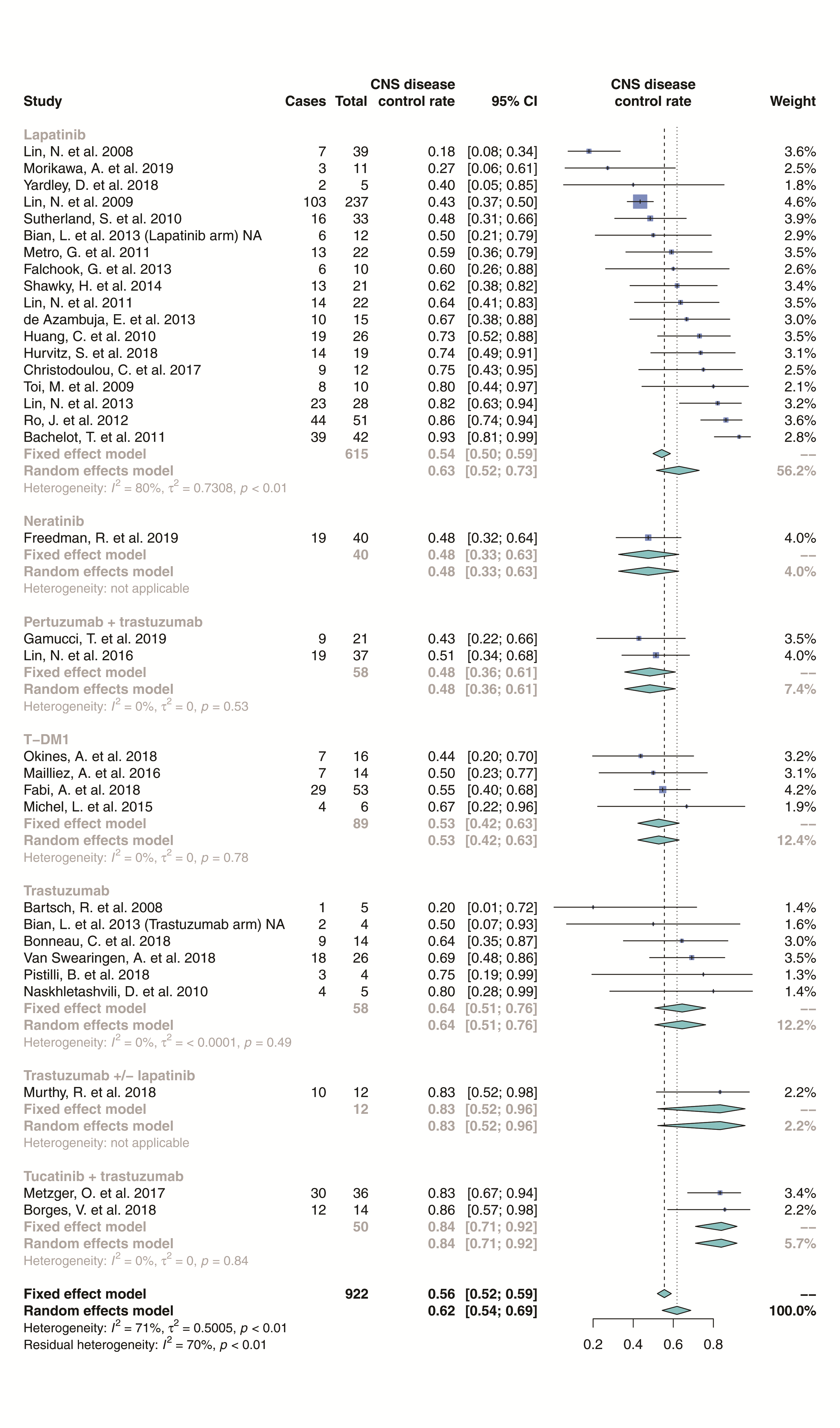


**Figure S4. Intracranial disease control rate in patients who received HER2-targeted therapy, stratified by publication before or after 2018.** Proportions for iDCR were extracted from eligible studies and pooled in meta-analysis. Studies here are stratified by publication year: before or during 2018 versus after. The size of each box represents the weight of each study in the meta-analysis. The vertical dashed and dotted lines represent the points of summary for fixed and random effects models, respectively, and the diamonds represent 95% CI for the summary proportions. Analyses were performed with the R programming language^11^ and the R package meta.^2^


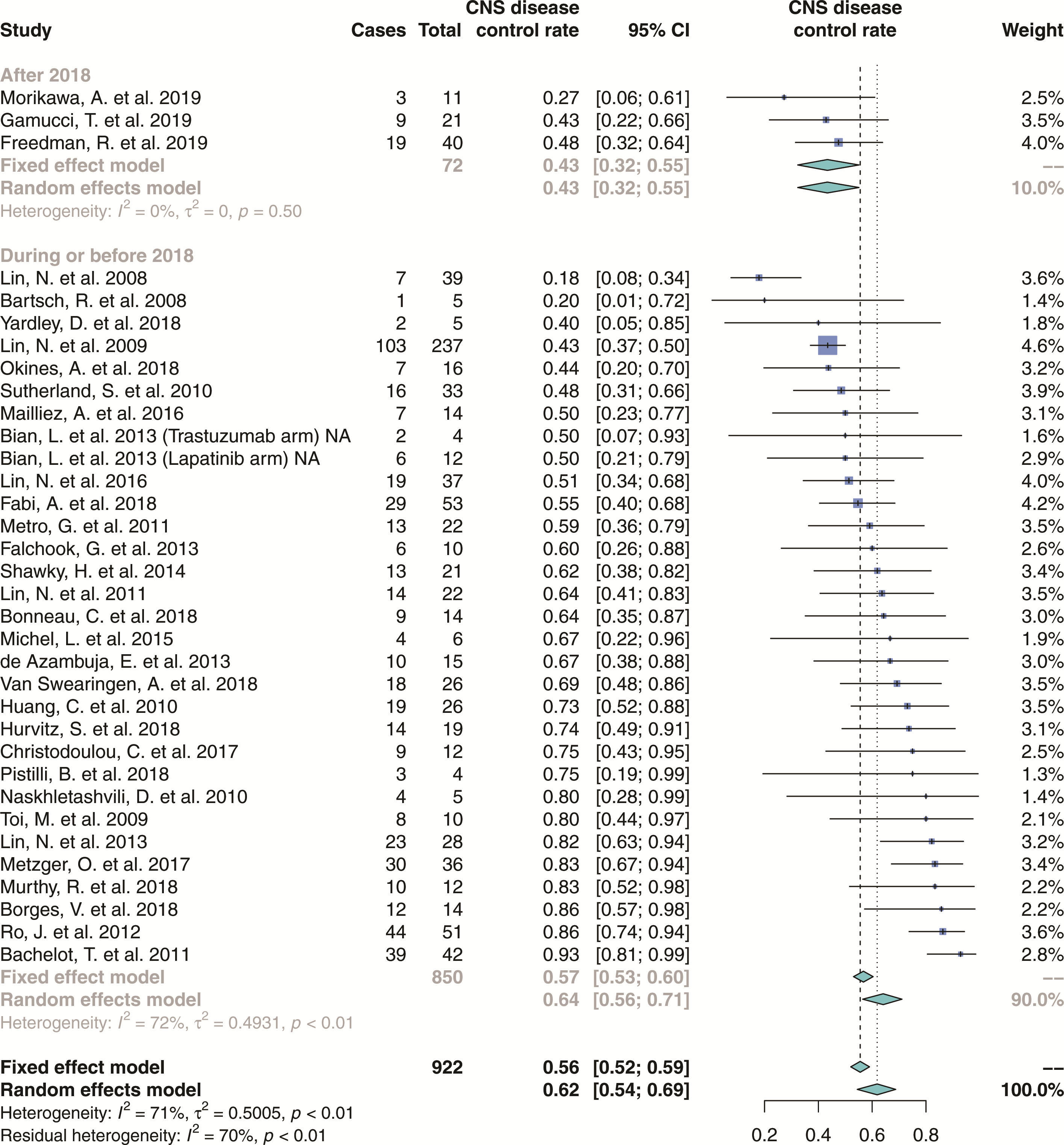


**Figure S5. Intracranial complete response rate in patients who received HER2-targeted therapy.** Proportions for iCRR were extracted from eligible studies and pooled in meta-analysis. Studies here are stratified by study design. The size of each box represents the weight of each study in the meta-analysis. The vertical dashed and dotted lines represent the points of summary for fixed and random effects models, respectively, and the diamonds represent 95% CI for the summary proportions. Analyses were performed with the R programming language^11^ and the R package meta.^2^


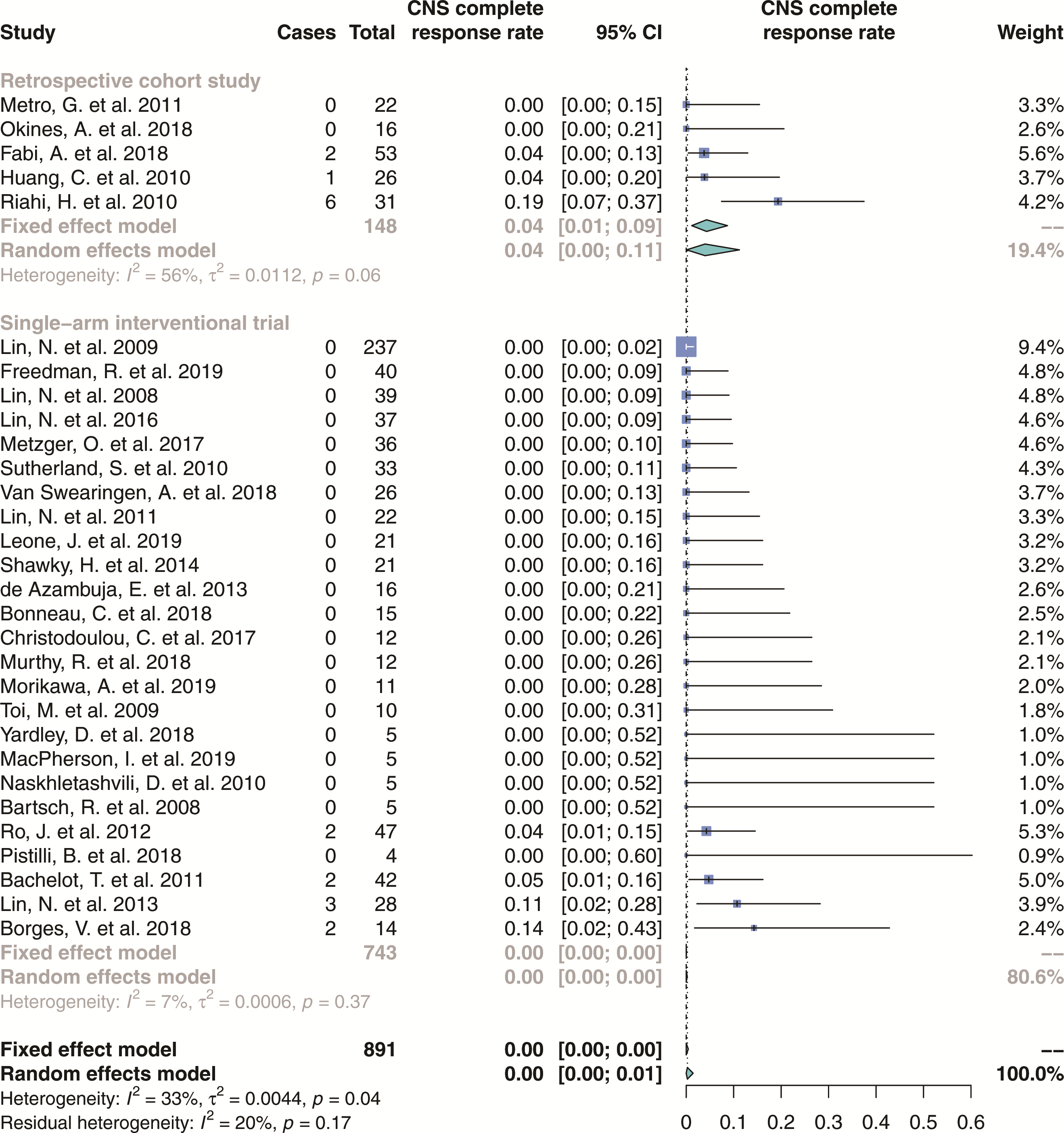


**Figure S6. Grade 3+ CTCAE adverse event rate in patients who received HER2-targeted therapy, stratified by drug structure.** Proportions for grade 3+ CTCAE adverse event rate were extracted from eligible studies and pooled in meta-analysis. Studies here are stratified by drug structure: antibody-based versus small-molecule inhibitors. Study regimens containing therapies in both classes were assigned to the small-molecule inhibitor stratum. The size of each box represents the weight of each study in the meta-analysis. The vertical dashed and dotted lines represent the points of summary for fixed and random effects models, respectively, and the diamonds represent 95% CI for the summary proportions. Analyses were performed with the R programming language^11^ and the R package meta.^2^


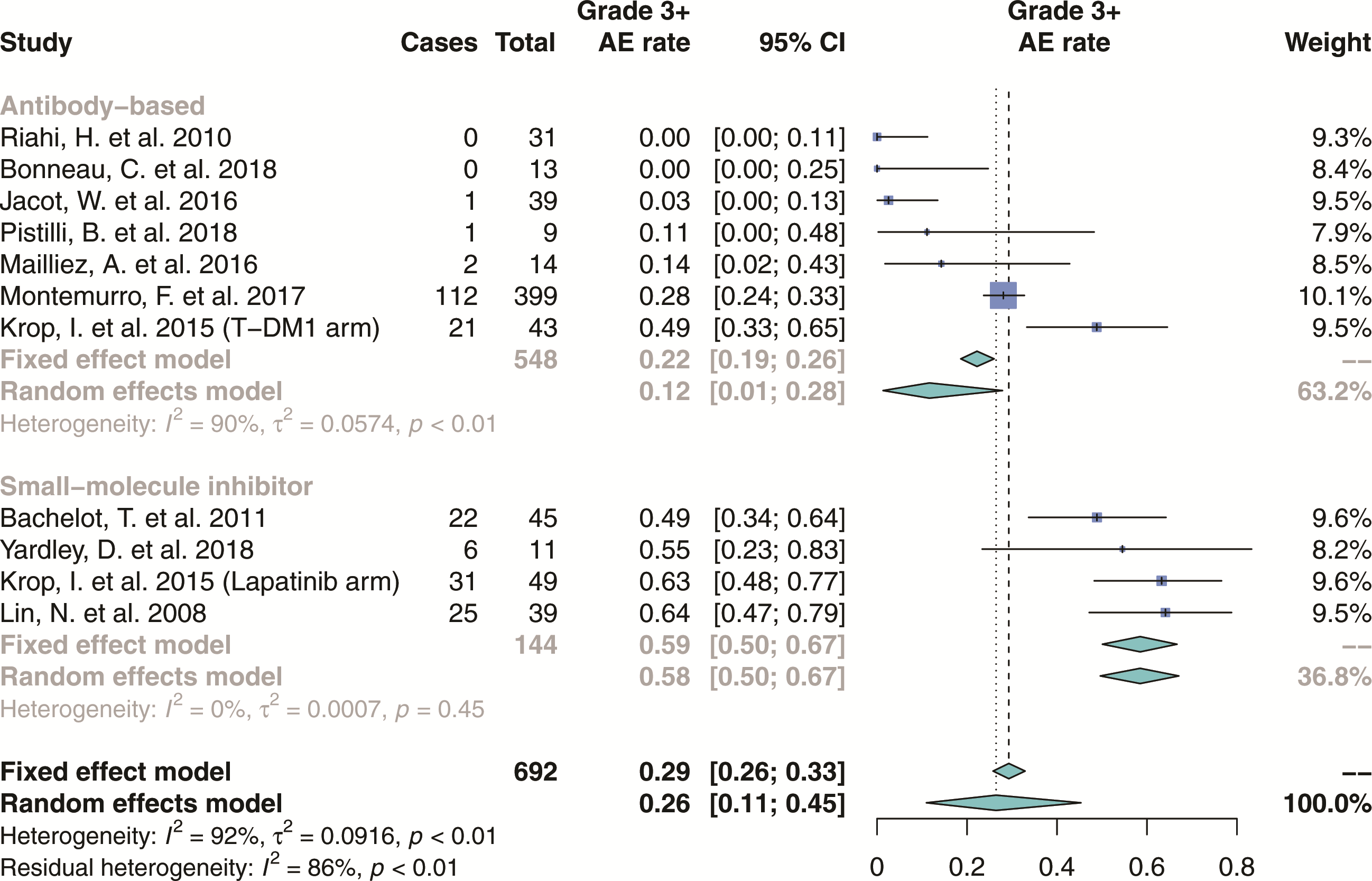


**Figure S7. Traffic light plot for risk of bias in interventional studies.** The Cochrane Risk of Bias 2 tool (RoB 2) was employed to assess risk of bias in interventional studies including RCTs and single-arm interventional trials. The traffic light plot displays overall and domain-specific risk of bias for individual studies. Figure generated with the R programming language^11^ and the R package robvis.^12^


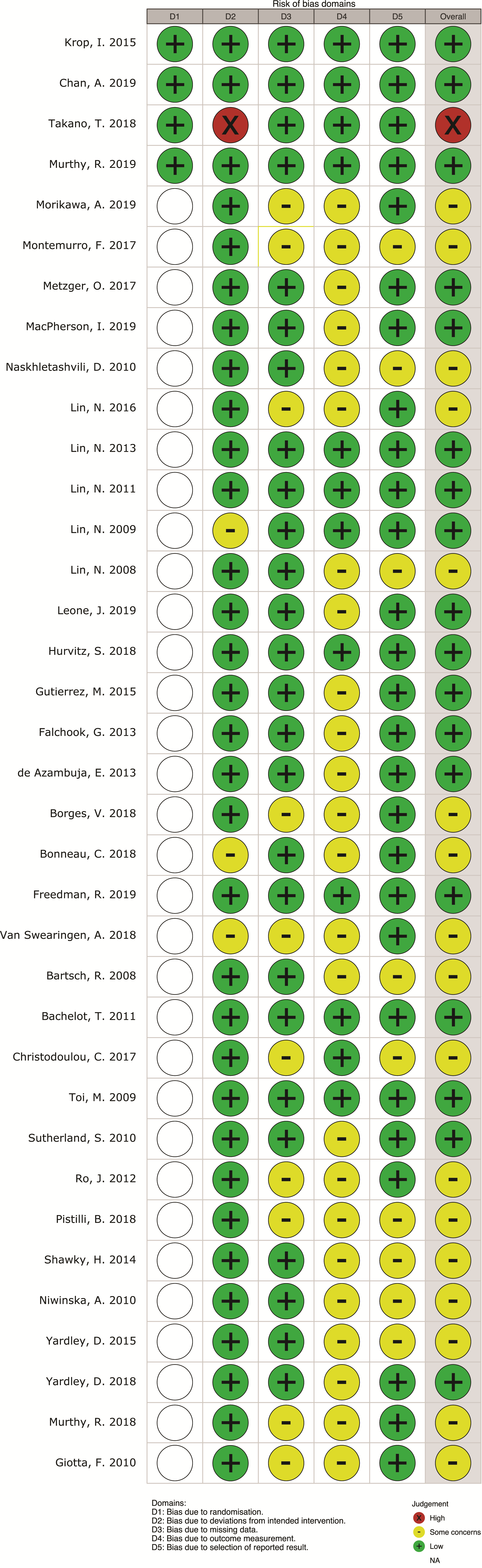


**Figure S8. Risk of bias summary plot for interventional studies.** The Cochrane Risk of Bias 2 tool (RoB 2) was employed to assess risk of bias in interventional studies including RCTs and single-arm interventional trials. The summary plot displays the distribution of overall and domain-specific risk of bias across individual studies. Figure generated with the R programming language^11^ and the R package robvis.^12^


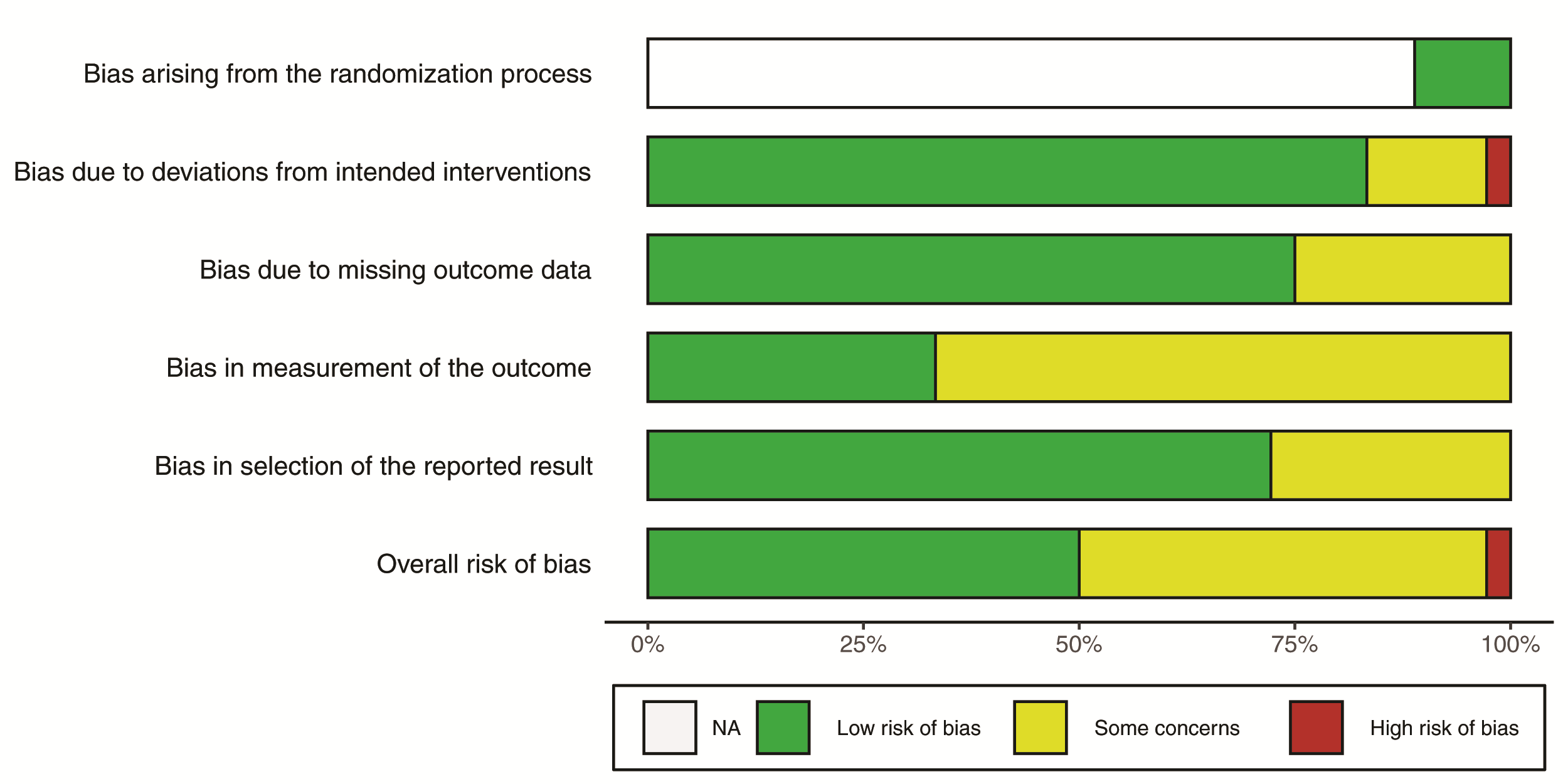


**Figure S9. Traffic light plot for risk of bias in observational studies.** The Newcastle-Ottawa Scale for cohort studies (NOS) was employed to assess risk of bias in observational studies including prospective and retrospective cohort studies. The traffic light plot displays overall and domain-specific risk of bias for individual studies. Figure generated with the R programming language^11^ and the R package robvis.^12^


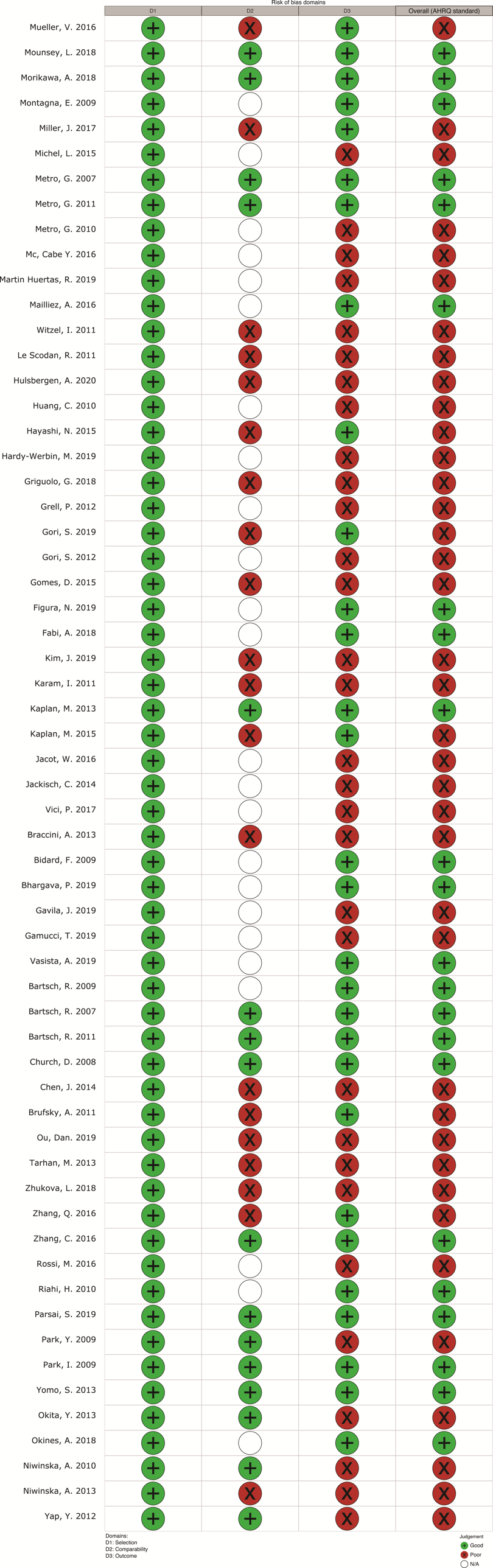


**Figure S10. Risk of bias summary plot for observational studies.** The Newcastle-Ottawa Scale for cohort studies (NOS) was employed to assess risk of bias in observational studies including prospective and retrospective cohort studies. The summary plot displays the distribution of overall and domain-specific risk of bias across individual studies. Figure generated with the R programming language^11^ and the R package robvis.^12^


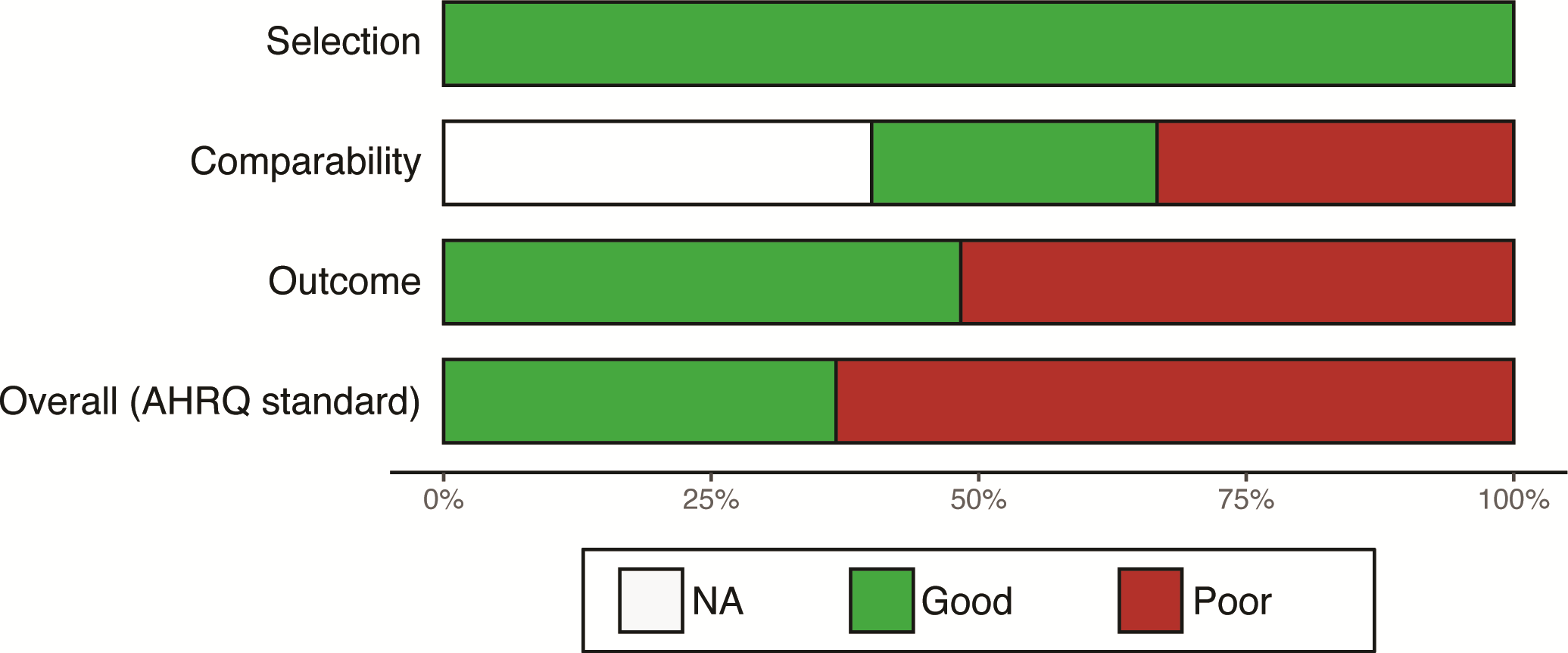


**Figure S11. Traffic light plot for risk of bias in non-randomized controlled trials.** The Risk Of Bias In Non-randomized Studies of Interventions (ROBINS-I) tool was employed to assess risk of bias in non-randomized controlled trials. One study with this design was included from the literature search after application of eligibility criteria. The traffic light plot displays overall and domain-specific risk of bias. Figure generated with the R programming language^11^ and the R package robvis.^12^


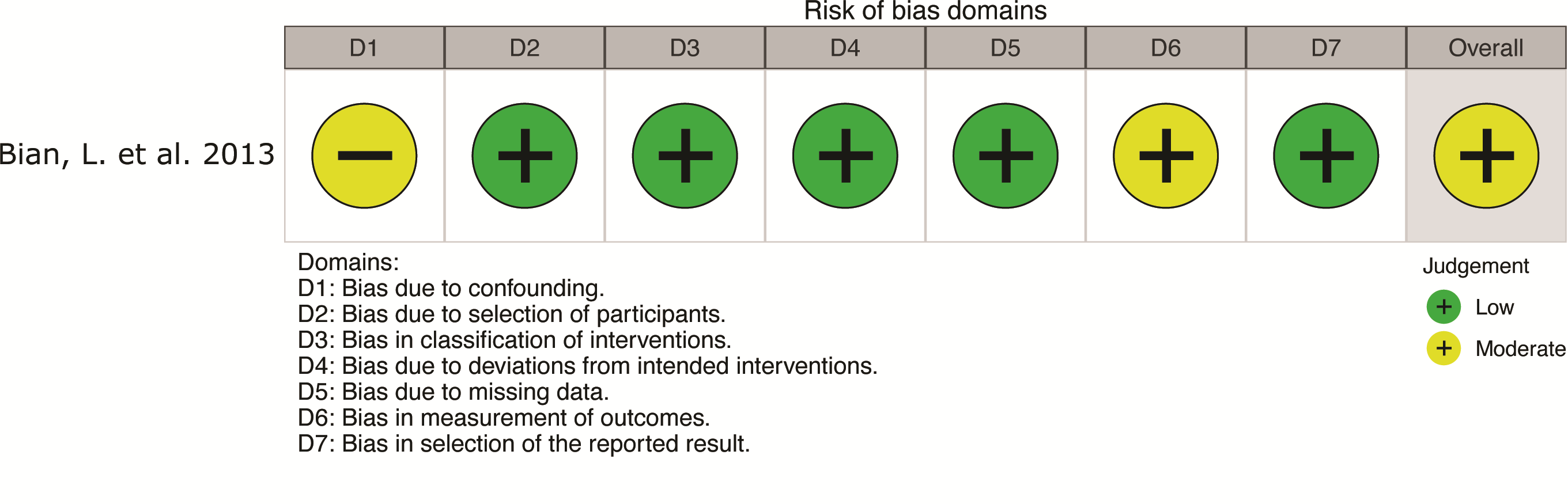


**Figure S12. Funnel plot for publication bias among studies reporting intracranial disease control rate.** In this plot, each grey dot represents one study. The white triangle represents the 90% confidence boundary centred around log-odds zero, corresponding to iDCR 50%. Dark grey, medium grey, and light grey represent the 90–95%, 95–99%, and 99%+ confidence regions around iDCR 50%. The solid and dotted vertical lines represent the random- and fixed-effects summary estimates, respectively. The diagonal dotted lines represent the 95% CI for the fixed-effects estimate. Egger’s test (p = .01) suggests potential publication bias. Figure generated with the R programming language^11^ and the R package meta.^2^


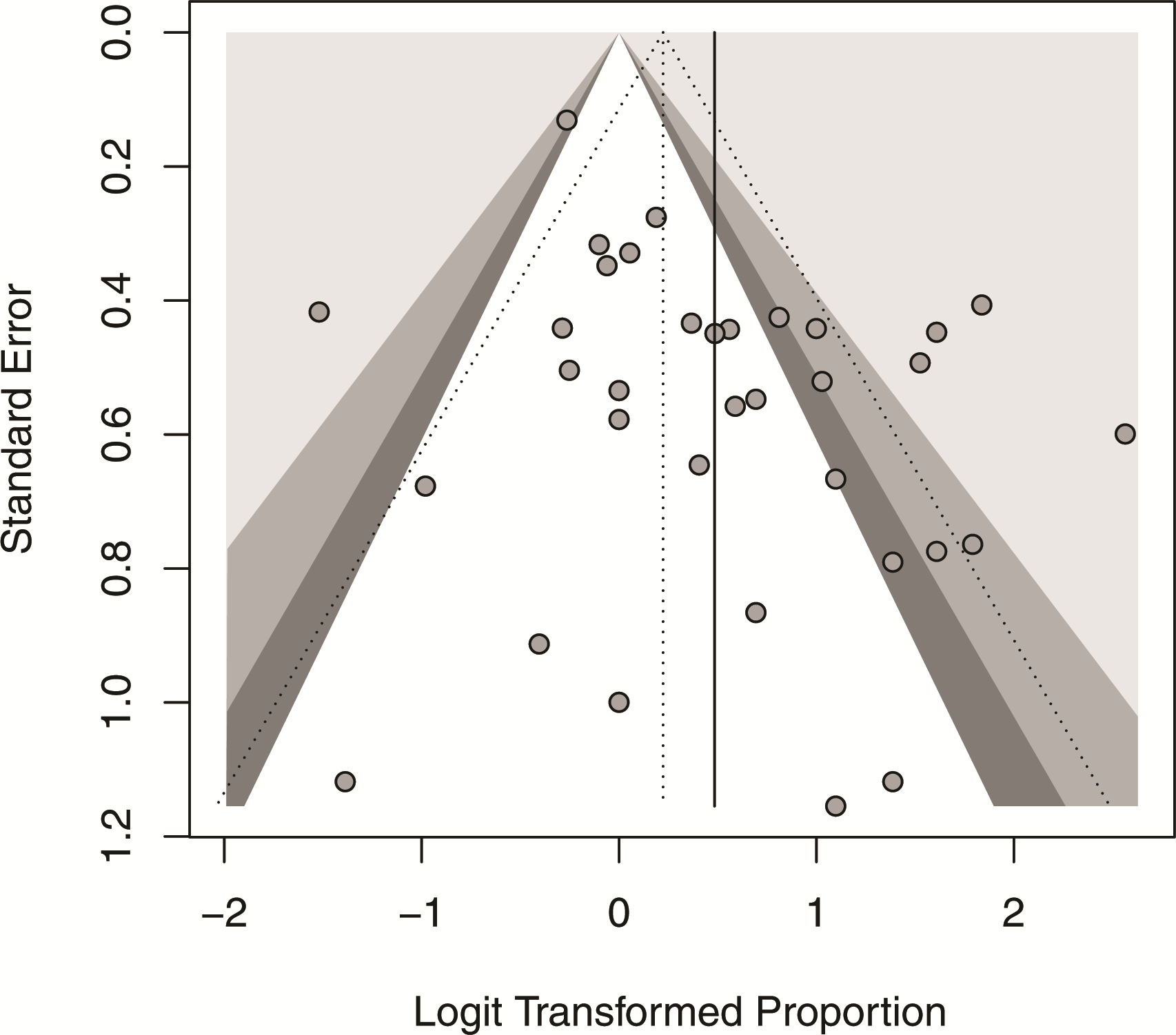


**Figure S13. Funnel plot for publication bias among studies reporting intracranial complete response rate.** In this plot, each grey dot represents one study. The white triangle represents the 90% confidence boundary centred around double arcsine-transformed zero. Dark grey, medium grey, and light grey represent the 90–95%, 95–99%, and 99%+ confidence regions around this point. The solid and dotted vertical lines represent the random- and fixed-effects summary estimates, respectively. The diagonal dotted lines represent the 95% CI for the fixed-effects estimate. Egger’s test (p = .02) suggests potential publication bias. Figure generated with the R programming language^11^ and the R package meta.^2^


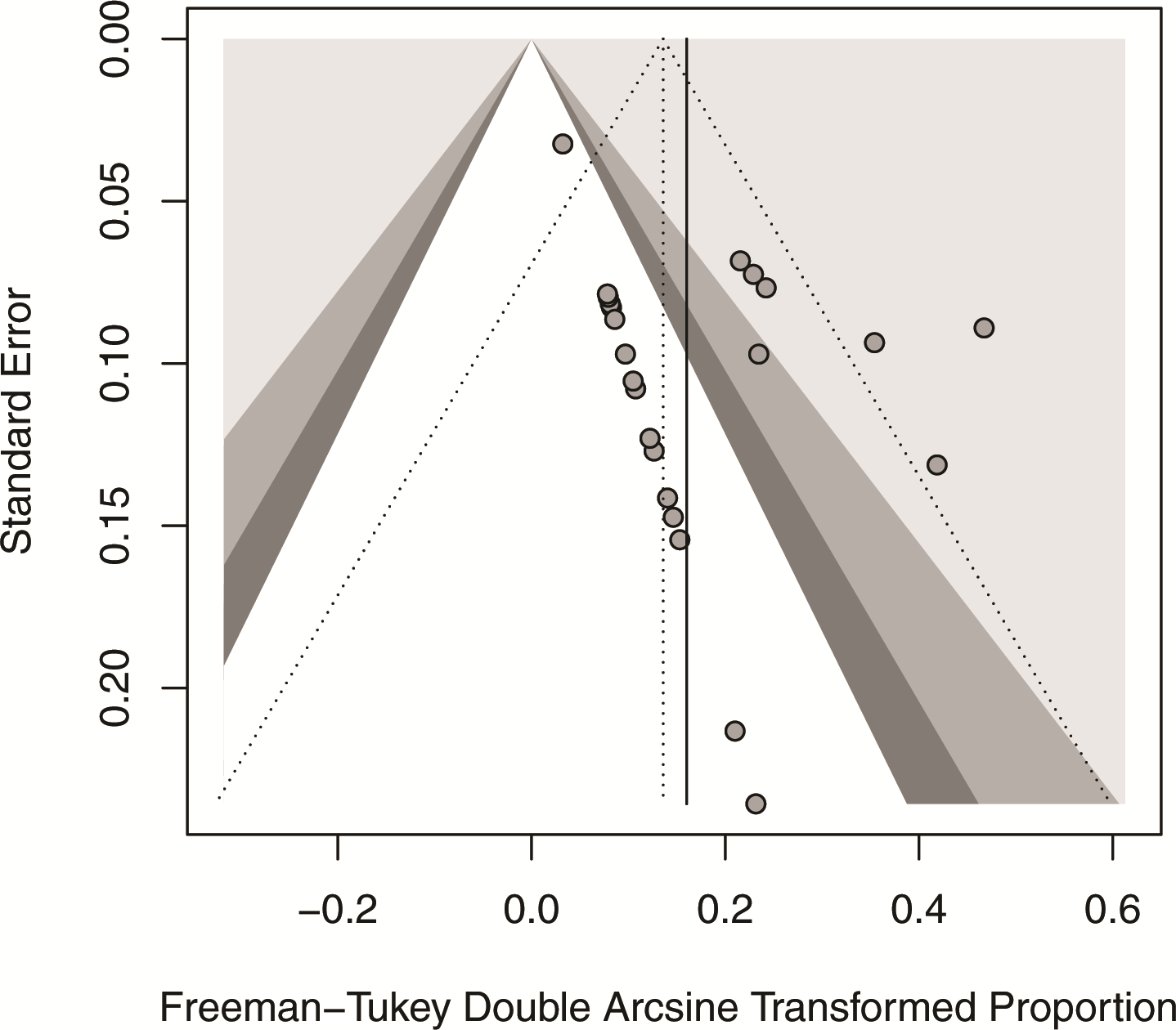


**Figure S14. Funnel plot for publication bias among studies reporting intracranial objective response rate.** In this plot, each grey dot represents one study. The white triangle represents the 90% confidence boundary centred around double arcsine-transformed zero. Dark grey, medium grey, and light grey represent the 90–95%, 95–99%, and 99%+ confidence regions around this point. The solid and dotted vertical lines represent the random- and fixed-effects summary estimates, respectively. The diagonal dotted lines represent the 95% CI for the fixed-effects estimate. Egger’s test (p = .70) fails to suggest potential publication bias. Figure generated with the R programming language^11^ and the R package meta.^2^


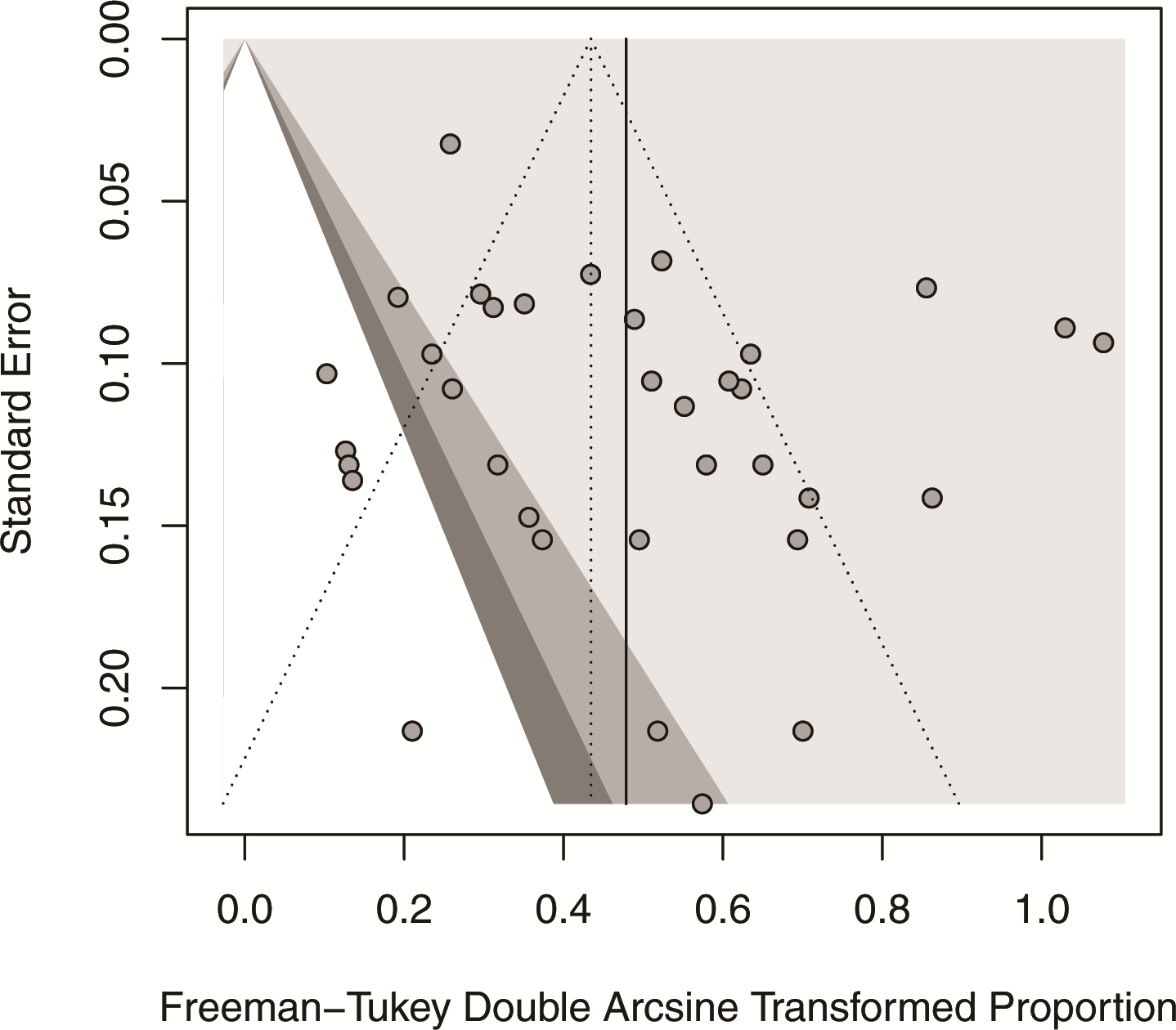


**Figure S15. Funnel plot for publication bias among studies reporting grade 3+ adverse event rate.** In this plot, each grey dot represents one study. The white triangle represents the 90% confidence boundary centred around double arcsine-transformed zero. Dark grey, medium grey, and light grey represent the 90–95%, 95–99%, and 99%+ confidence regions around this point. The solid and dotted vertical lines represent the random- and fixed-effects summary estimates, respectively. The diagonal dotted lines represent the 95% CI for the fixed-effects estimate. Egger’s test (p = .87) fails to suggest potential publication bias. Figure generated with the R programming language^11^ and the R package meta.^2^


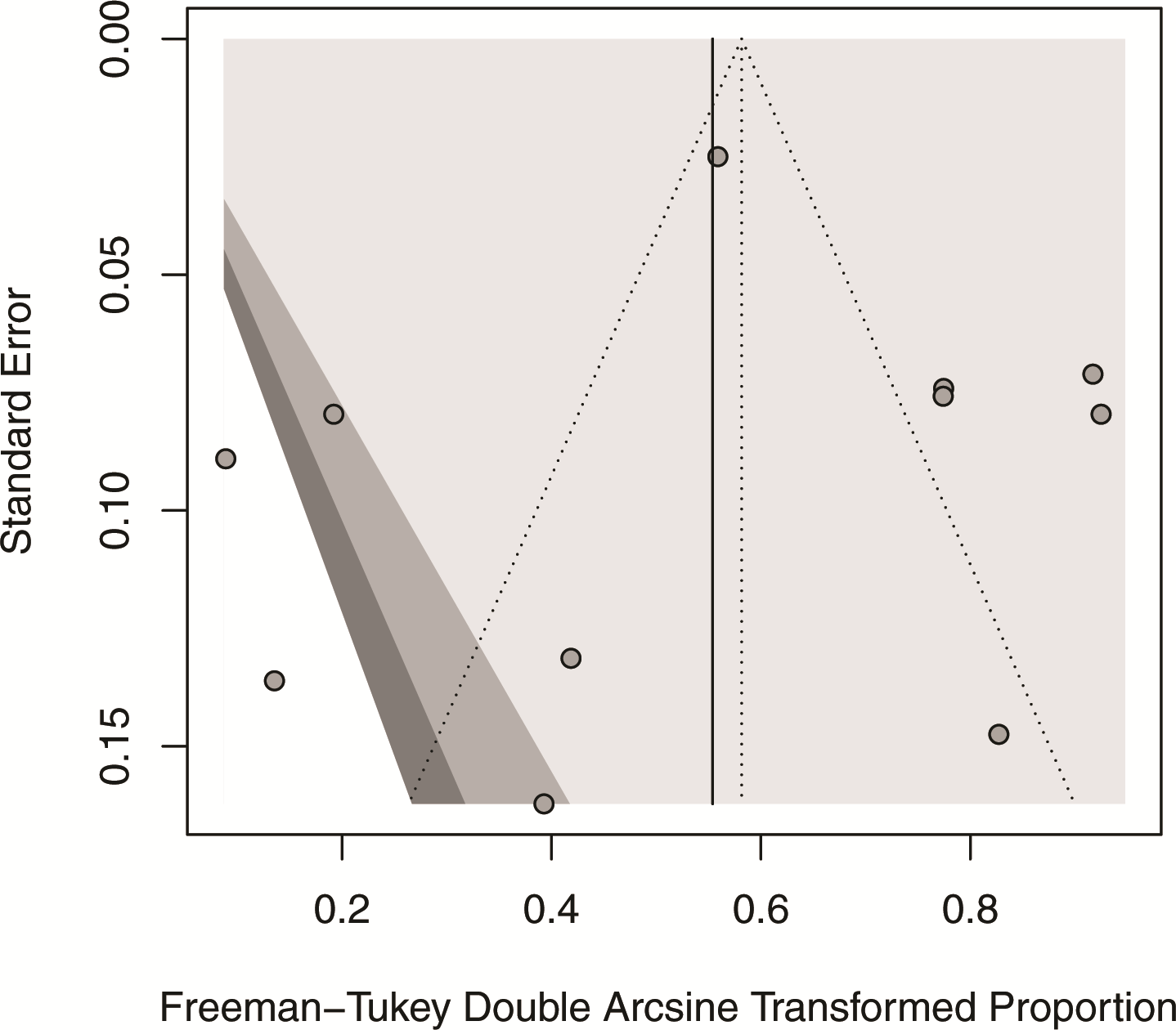


**Figure S16. Funnel plot for publication bias among studies reporting overall survival.** In this plot, each grey dot represents one study. The white triangle represents the 90% confidence boundary centred around the point of equivalence. Dark grey, medium grey, and light grey represent the 90–95%, 95–99%, and 99%+ confidence regions around this point. The solid and dotted vertical lines represent the random- and fixed-effects summary estimates, respectively. The diagonal dotted lines represent the 95% CI for the fixed-effects estimate. Egger’s test (p = .75) fails to suggest potential publication bias. Figure generated with the R programming language^11^ and the R package meta.^2^


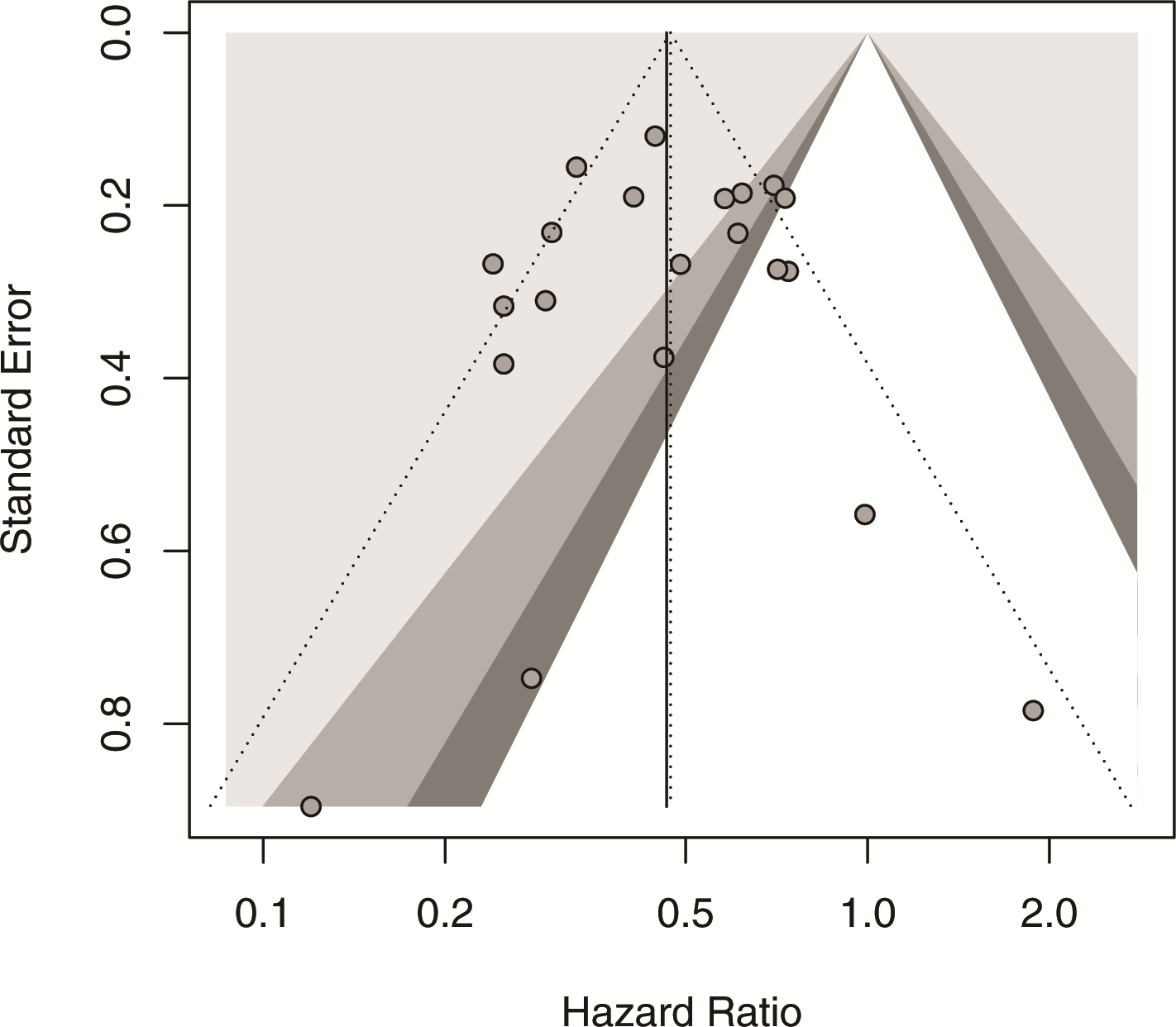


**Figure S17. Funnel plot for publication bias among studies reporting progression-free survival.** In this plot, each grey dot represents one study. The white triangle represents the 90% confidence boundary centred around the point of equivalence. Dark grey, medium grey, and light grey represent the 90–95%, 95–99%, and 99%+ confidence regions around this point. The solid and dotted vertical lines represent the random- and fixed-effects summary estimates, respectively. The diagonal dotted lines represent the 95% CI for the fixed-effects estimate. Egger’s test was deferred due to the number of studies (n = 4). Figure generated with the R programming language^11^ and the R package meta.^2^


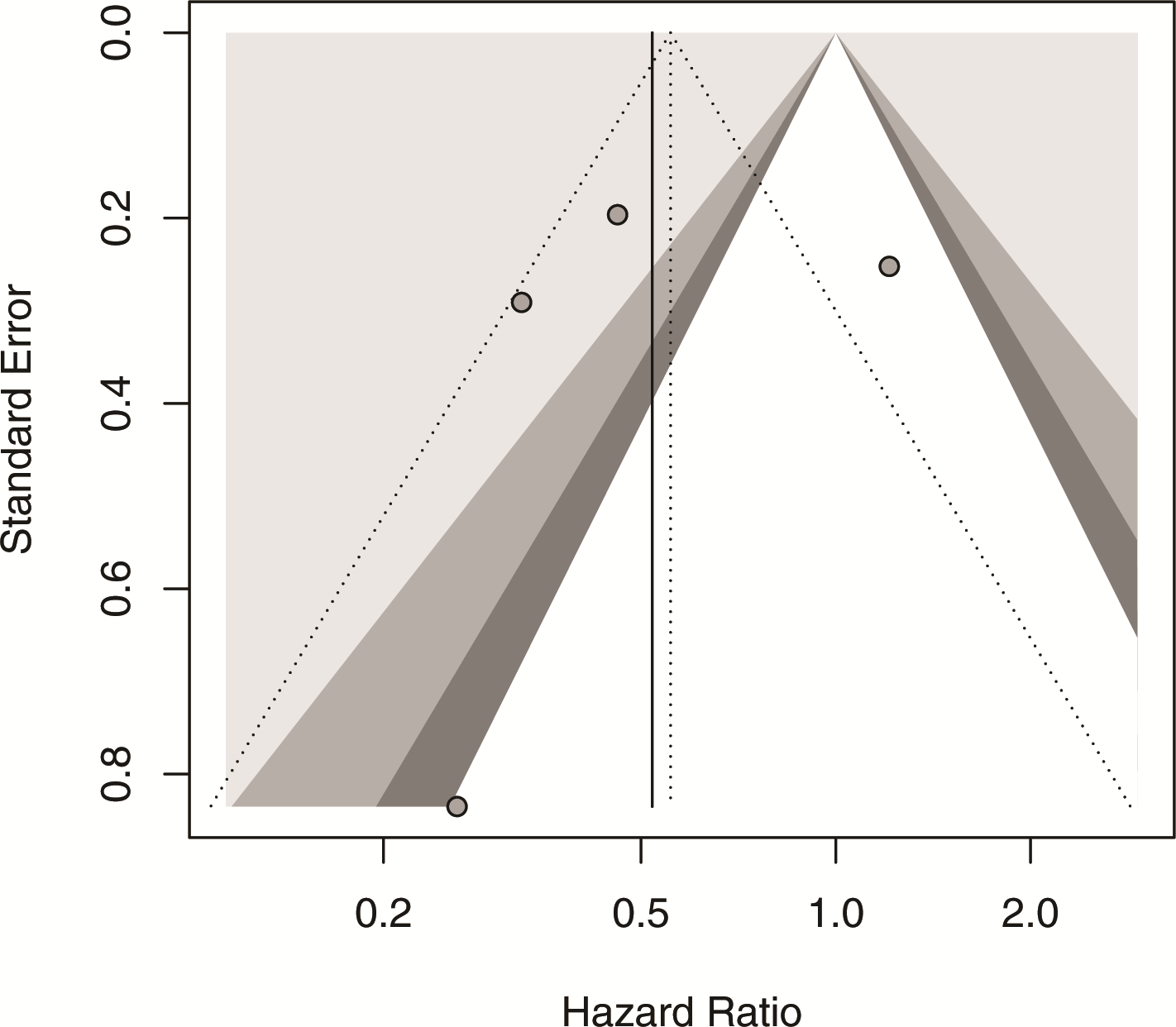


## References

**1.** Moher D, Liberati A, Tetzlaff J, Altman DG, Group P. Preferred reporting items for systematic reviews and meta-analyses: the PRISMA statement. *J Clin Epidemiol.* 2009; 62(10):1006-1012.

**2.** Schwarzer G. meta: an R package for meta-analysis. *R News.* 2007;7(3):40–45. <https://cran.r-project.org/doc/Rnews/Rnews_2007-3.pdf>.

**3.** Higgins J, Thomas J, Chandler J, et al. (editors).  C*ochrane Handbook for Systematic Reviews of Interventions*  version 6.0 (updated July 2019). Cochrane, 2019. Available from <www.training.cochrane.org/handbook>. Accessed July 5th, 2020.

**4.** Guyot P, Ades AE, Ouwens MJ, Welton NJ. Enhanced secondary analysis of survival data: reconstructing the data from published Kaplan-Meier survival curves. *BMC Med Res Methodol.* 2012; 12:9.

**5.** Parsai S, Miller JA, Juloori A, et al. Stereotactic radiosurgery with concurrent lapatinib is associated with improved local control for HER2-positive breast cancer brain metastases. *J Neurosurg.* 2019:1-9.

**6.** Higgins J, Thomas J, Chandler J, et al. *Cochrane Handbook for Systematic Reviews of Interventions*  version 6.0 (updated July 2019). Cochrane, 2019. Available from <www.training.cochrane.org/handbook>. Accessed July 5th, 2020.

**7.** Michael Borenstein, L. V. Hedges, J. P. T. Higgins and H. R. Rothstein.  Introduction to Meta-Analysis.  © 2009 John Wiley & Sons, Ltd. ISBN: 978-0-470-05724-7.

**8.** Sperduto PW, Kased N, Roberge D, et al. Summary report on the graded prognostic assessment: an accurate and facile diagnosis-specific tool to estimate survival for patients with brain metastases. *J Clin Oncol.* 2012; 30(4):419-425.

**9.** GRADEpro GDT: GRADEpro Guideline Development Tool [Software]. McMaster University, 2015 (developed by Evidence Prime, Inc.). Available from  gradepro.org . Accessed July 5th, 2020.

**10.** Guyatt GH, Oxman AD, Vist GE, et al. GRADE: an emerging consensus on rating quality of evidence and strength of recommendations. *BMJ.* 2008; 336(7650):924-926.

**11.** Team RC. R: a language and environment for statistical computing. 2019. <https://www.R-project.org/>.

**12.** McGuinness LA (2019). “robvis: An R package and web application for visualising risk-of-bias assessments.” URL:  <https://github.com/mcguinlu/robvis> .
